# YOLOv12-BDA: A Dynamic Multi-Scale Architecture for Small Weed Detection in Sesame Fields

**DOI:** 10.3390/s25226927

**Published:** 2025-11-13

**Authors:** Guofeng Xia, Xin Li

**Affiliations:** School of Mechanical Engineering, Chongqing Three Gorges University, Chongqing 404100, China; yudsjmoon@163.com

**Keywords:** YOLOv12, weed detection, small-object detection, deep learning, precision agriculture, dynamic multi-scale, sesame fields

## Abstract

Sesame (*Sesamum indicum* L.) is one of the most important oilseed crops globally, valued for its high content of unsaturated fatty acids, proteins, and essential nutrients. However, weed infestation represents a major constraint on sesame productivity, competing for resources and releasing allelopathic compounds that can significantly reduce both yield and quality without timely control. To address the challenge of low detection accuracy in complex agricultural environments with dense weed distributions, this study proposes YOLOv12-BDA, a dynamic multi-scale architecture for small weed detection in sesame fields. The proposed architecture incorporates three key dynamic innovations: (1) an Adaptive Feature Selection (AFS) dual-backbone network with a Dynamic Learning Unit (DLU) module that enhances cross-branch feature extraction while reducing computational redundancy; (2) a Dynamic Grouped Convolution and Channel Mixing Transformer (DGCS) module that replaces the C3K2 component to enhance real-time detection of small weeds against complex farmland backgrounds; and (3) a Dynamic Adaptive Scale-aware Interactive (DASI) module integrated into the neck network to strengthen multi-scale feature fusion and detection accuracy. Experimental validation on high-resolution sesame field datasets demonstrates that YOLOv12-BDA significantly outperforms baseline models. The proposed method achieves mAP@50 improvements of 6.43%, 11.72%, 7.15%, 5.33%, and 4.67% over YOLOv5n, YOLOv8n, YOLOv10n, YOLOv11n, and YOLOv12n, respectively. The results confirm that the proposed dynamic architecture effectively improves small-target weed detection accuracy at the cost of increased computational requirements (4.51 M parameters, 10.7 GFLOPs). Despite these increases, the model maintains real-time capability (113 FPS), demonstrating its suitability for precision agriculture applications prioritizing detection quality. Future work will focus on expanding dataset diversity to include multiple crop types and optimizing the architecture for broader agricultural applications.

## 1. Introduction

Sesame (*Sesamum indicum* L.) is one of China’s most important oilseed crops and is traditionally considered one of the country’s “Eight Grains” due to its high nutritional and economic value. Rich in unsaturated fatty acids, high-quality proteins, and essential trace elements, sesame plays a vital role in the edible oil industry and serves as a critical raw material for functional food development [1,2,3,4]. However, weed infestation represents a major constraint on sesame productivity. Weeds compete for light, soil moisture, and nutrients while simultaneously releasing allelopathic compounds that inhibit sesame growth. Without proper and timely control, weed overgrowth can significantly reduce both yield and quality [5]. Traditional weeding methods rely heavily on manual labor and farmer experience. While effective, these approaches are labor-intensive, costly, and unsuitable for the large-scale production [6]. Therefore, accurate and cost-effective weed detection has become crucial for sustainable sesame cultivation and precision agriculture advancement.

Weed detection in sesame fields presents distinct challenges that require specialized approaches beyond those developed for other crops. First, sesame seedlings exhibit high visual similarity to several dominant broadleaf weed species during early growth stages, particularly during the 2–4 week period following emergence. Common weed species such as Amaranthus spp. (pigweed) and Portulaca oleracea (purslane) share comparable leaf morphologies, green spectral signatures, and spatial configurations with young sesame plants, complicating automated discrimination through conventional color-based or shape-based feature extraction methods [7,8]. Second, sesame cultivation typically employs dense planting configurations with narrow row spacing, generally ranging from 30 to 45 cm for optimal yield performance, as demonstrated in recent agronomic optimization studies [9]. These dense planting patterns result in substantial inter-plant occlusion, where overlapping foliage from adjacent plants partially obscures target boundaries, creating additional challenges for accurate object localization and boundary delineation in detection systems [10]. Third, the critical intervention window for effective weed control in sesame has been established through field studies as occurring between 15 and 35 days after sowing, during which both crop and weed species are at similar developmental stages with minimal morphological differentiation [11]. This temporal constraint necessitates early-stage detection capabilities when visual discrimination is most challenging due to the morphological convergence between crop and weed seedlings. These combined factors—morphological similarity, dense spatial configurations, and temporal constraints—collectively necessitate detection architectures specifically tailored to sesame production systems rather than direct transfer of methods proven effective in other crop environments.

Recent advances in computer vision and deep learning have revolutionized intelligent weed management. Object detection algorithms are generally categorized into two types. The first category comprises two-stage detectors (R-CNN [12], Fast R-CNN [13], Faster R-CNN [14]) that achieve high accuracy but have slower inference speeds, while the second includes single-stage detectors (YOLO [15,16], SSD [17], RetinaNet [18]) that offer faster detection with slightly reduced accuracy. The YOLO series achieves an optimal balance between real-time performance and accuracy through its one-stage regression framework, making it widely adopted for applications requiring rapid detection.

The YOLO architecture has undergone continuous evolution across multiple versions, each introducing distinct architectural innovations to address specific detection challenges while presenting inherent trade-offs. YOLOv5 [19] established the foundation with the CSPDarknet backbone and C3 modules for cross-stage feature fusion, employing anchor-based detection using predefined anchor boxes for multi-scale object localization. However, the anchor-based approach requires manual design of anchor dimensions and ratios, which may not generalize optimally across diverse datasets without dataset-specific tuning [20,21]. YOLOv8 [22] transitioned to an anchor-free paradigm, eliminating manual anchor design through directly predicting object centers and dimensions, while introducing C2f modules that enhanced gradient flow. Although this simplifies the detection pipeline, anchor-free methods can face challenges in precise localization for small objects where explicit scale priors provide valuable inductive bias [23]. YOLOv10 [24] advanced efficiency through architectural decoupling, separating classification and localization heads while implementing NMS-free training. This decoupling, while reducing redundant computations, introduces multiple optimization pathways that may increase training complexity [24]. YOLOv11 [25] incorporates enhanced backbone and neck architectures for improved feature extraction capabilities. Despite these enhancements, it maintains static feature fusion strategies that apply uniform processing regardless of target scale distribution within images [26]. Most recently, YOLOv12 [27] represents a paradigm shift toward attention-centric design, incorporating Area Attention (A2) modules throughout the backbone. While attention mechanisms excel at modeling long-range dependencies, they may introduce computational overhead and can potentially overlook fine-grained local features that convolutional operations naturally capture [27,28]. This architectural progression reflects the field’s evolution from handcrafted anchor-based designs to learnable attention-driven feature extraction, with each iteration addressing specific limitations in detection accuracy, computational efficiency, or deployment flexibility, yet also introducing new challenges that must be considered for specific application domains.

Despite extensive application of YOLO-based detection frameworks across diverse agricultural scenarios, research specifically addressing weed detection in sesame cultivation systems remains notably limited. Recent studies have begun exploring deep learning approaches for sesame field applications, with promising initial results demonstrating the feasibility of automated detection in this crop system. Naik and Chaubey demonstrated that region-based convolutional neural networks could achieve 96.84% detection accuracy when identifying and classifying weed species in sesame crops, effectively distinguishing between crop plants and multiple weed categories through supervised learning on annotated field imagery [7]. Similarly, Chen et al. developed an enhanced YOLO-based detection model incorporating attention mechanisms and adaptive spatial feature fusion strategies specifically optimized for sesame field conditions, achieving F1 scores of 0.91 for sesame plant detection and 0.92 for weed identification [8]. While recent YOLO-based methods have demonstrated effectiveness in other crop systems [20,21,23], these approaches do not directly address the unique challenges specific to sesame cultivation environments. However, these pioneering studies primarily focused on establishing proof-of-concept for deep learning applicability in sesame systems rather than addressing the specific architectural challenges posed by real-time small-target detection in complex field environments characterized by varying illumination conditions, dense spatial planting patterns, and extreme within-image scale heterogeneity [10,29]. The unique combination of morphological similarity during critical early growth stages, variable planting configurations across different geographical regions and management practices, and the imperative for early-stage intervention during narrow temporal windows necessitates specialized architectural innovations that extend beyond simple adaptation of detection frameworks proven effective in other crop systems with fundamentally different visual characteristics and agronomic requirements.

Despite these advances, research on weed detection specifically for sesame fields remains limited, with most studies focusing on other crops or controlled imaging conditions. In practical field environments, automated weed detection in sesame cultivation systems presents three interconnected technical challenges that demand specialized architectural solutions:

First, extreme small-object detection under substantial scale heterogeneity. During the optimal intervention window for weed control (15–35 days post-sowing, as established by agronomic studies [11]), early-stage weed seedlings occupy minimal spatial footprints in field images captured at standard agronomic monitoring heights. According to the widely adopted Common Objects in Context (COCO) dataset benchmark standards for object detection, small objects are formally defined as those occupying less than 32 × 32 pixels in area, or equivalently covering less than approximately 0.1% of a typical high-resolution image [30]. Agricultural small object detection research has demonstrated that targets falling within this scale range present substantial challenges for conventional detection networks, as the progressive spatial resolution degradation through successive down sampling operations in deep convolutional architectures compromises the preservation of discriminative features necessary for accurate localization. The challenge is further exacerbated in sesame field scenarios because individual images simultaneously contain targets spanning multiple distinct scale categories: early-stage weed seedlings approaching the COCO small object threshold, intermediate-sized sesame plants at various developmental stages, and mature weed specimens that may exceed crop plant dimensions. Traditional single-scale feature extraction mechanisms demonstrate fundamental limitations when confronted with such extreme heterogeneity: shallow network layers preserve the high spatial resolution necessary for detecting small objects but lack sufficient semantic abstraction capability to discriminate between morphologically convergent crop and weed features, while progressively deeper layers develop increasingly discriminative semantic representations but irreversibly lose fine-grained spatial localization information through successive pooling operations.

Second, the imperative for adaptive multi-scale feature fusion mechanisms. Traditional Feature Pyramid Network (FPN) architectures employ static fusion strategies—typically implementing fixed element-wise addition operations such as *F*_out = *F*_high + *F*_low, or predetermined concatenation schemes with uniform weighting—that process all spatial locations identically regardless of the local scale characteristics of objects present at different image regions [26]. This architectural design operates under the implicit assumption that features from different pyramid levels contribute equally to detection performance across all input scenarios and all spatial locations within images. However, this assumption proves problematic in agricultural scenarios where individual field images simultaneously contain targets exhibiting dramatically different scale requirements: small early-stage weeds that primarily benefit from fine-grained high-resolution spatial features for precise boundary localization, medium-sized sesame plants that require balanced integration of spatial and semantic information, and large mature weeds that predominantly require high-level semantic context from deep network layers for robust classification. The inability to adaptively adjust fusion weights based on target-specific local characteristics results in suboptimal feature representations, where different object sizes receive non-optimal feature combinations that fail to leverage the complementary strengths of multi-scale pyramid features.

Third, morphological similarity during critical growth stages demands highly discriminative feature extraction. Sesame seedlings and dominant broadleaf weed species exhibit convergent phenotypes during early developmental phases, sharing similar green spectral signatures, comparable leaf morphologies, and equivalent spatial configurations [7,8]. Reliable discrimination requires extraction of subtle visual differences including fine-grained leaf venation patterns, marginal serration characteristics, and early-stage growth trajectory variations—discriminative features that occupy minimal spatial footprints within high-resolution agricultural imagery. These challenges are compounded by substantial background variability characteristic of field environments: diverse soil surface textures, presence of crop residues and organic matter creating visual clutter, and illumination variations producing dramatic intra-field heterogeneity.

To address these challenges, this paper proposes YOLOv12-BDA, an improved YOLOv12 architecture specifically designed for small weed detection in sesame fields. Three synergistic architectural modifications are introduced to overcome the limitations of baseline YOLOv12 in agricultural scenarios.

- First, the single-backbone structure of YOLOv12 is replaced with an Adaptive Feature Selection (AFS) dual-backbone architecture incorporating HGStem and HGBlock modules for complementary spatial and semantic feature extraction. A Dynamic Learning Unit (DLU) is introduced to perform content-adaptive fusion of dual-backbone features through dynamically generated weights, addressing the challenge of simultaneously extracting fine-grained spatial details for small weed localization and high-level semantic features for robust crop-weed discrimination.
- Second, the C3K2 module in the YOLOv12 backbone is replaced with a novel Dynamic Grouped Convolution and Channel Mixing Transformer (DGCS) module. This module employs a 1:3 channel splitting ratio combined with depthwise grouped convolution and systematic channel reshuffling to reduce computational overhead while enhancing cross-channel information exchange, which is critical for distinguishing subtle morphological differences between visually similar crop and weed seedlings.
- Third, the standard PANet neck network is replaced with a Dynamic Adaptive Scale-aware Interactive (DASI) module that implements scale-adaptive dynamic weighting for multi-scale feature fusion. Unlike conventional feature pyramid networks that employ fixed element-wise addition, DASI computes fusion weights dynamically based on input feature activation patterns, enabling adaptive emphasis on different feature sources based on target characteristics within the same image.

These architectural innovations collectively address the specific challenges of sesame field weed detection—small-target size, extreme scale variation, and complex agricultural backgrounds. While the modifications increase computational cost relative to baseline, the architecture maintains real-time capability, making it suitable for agricultural platforms prioritizing enhanced detection accuracy.

## 2. Materials and Methods

### 2.1. Overview of YOLOv12

YOLOv12 is a recent real-time object detection algorithm that integrates attention mechanisms as core architectural components, contrasting with the predominantly CNN-based structures of previous YOLO versions. This design enables improved detection accuracy while maintaining real-time processing capabilities. Compared to its predecessors, YOLOv12 introduces three key architectural advancements. First, unlike YOLOv5-v8, which primarily employ convolutional operations with limited attention augmentation, YOLOv12 incorporates Area Attention (A2) modules throughout the backbone network. This design enhances global context modeling capabilities, which is particularly relevant for distinguishing small-targets from complex backgrounds in agricultural scenarios. Second, the A2 module reduces computational complexity by partitioning feature maps into four localized regions for attention computation, rather than performing global attention across entire feature maps. This approach maintains large receptive fields while reducing memory and computational requirements compared to standard self-attention mechanisms employed in vision transformers. Third, YOLOv12 employs Residual Efficient Layer Aggregation Networks (R-ELAN) with scaled residual connections (scaling factor 0.01) to address gradient optimization challenges in deep attention-based architectures. This differs from the CSPDarknet backbone in YOLOv5-v8 and the C2f modules in YOLOv11, potentially improving training stability and feature representation capability.

The YOLOv12 architecture comprises four primary components. The backbone network employs convolutional layers inherited from YOLOv11 in initial stages for basic feature extraction, while subsequent stages integrate A2 modules and R-ELAN structures for deep feature learning. The A2 module partitions input feature maps into four regions through vertical or horizontal division for localized attention computation, reducing computational complexity while preserving receptive field coverage. The neck network implements a feature pyramid architecture that performs multi-scale feature fusion, integrating semantic information from deep layers with spatial details from shallow layers to support detection across varying object scales. The detection head utilizes depthwise separable convolutions to output bounding box coordinates, classification scores, and confidence predictions. Additionally, Flash Attention implementation optimizes memory access patterns during attention computation, reducing memory overhead compared to standard attention implementations.

The architectural characteristics of YOLOv12 address specific challenges in sesame field weed detection. The attention mechanism’s ability to model long-range dependencies supports differentiation between visually similar crop and weed features. The multi-scale feature fusion capability is relevant for detecting weeds with significant size variation, from small seedlings to mature plants. Efficient memory utilization enables processing of high-resolution agricultural imagery (512 × 512 pixels in this study) without excessive computational resource requirements. According to the original study, YOLOv12 demonstrated superior performance in small object detection tasks compared to previous YOLO versions, with particular improvements in feature representation capability through its attention-centric design. These characteristics make it a suitable baseline architecture for the enhancements proposed in this study, specifically targeting improved weed detection accuracy in complex agricultural environments.

It should be noted that each YOLO version presents architectural trade-offs that affect performance in agricultural applications. The evolution from YOLOv5’s anchor-based detection to YOLOv8’s anchor-free paradigm represents a fundamental shift in object localization strategy, each with distinct advantages and limitations for small-target detection [22]. Similarly, the progression from predominantly convolutional architectures (YOLOv5-v11) to attention-centric designs (YOLOv12) reflects the ongoing exploration of optimal feature extraction mechanisms for diverse detection scenarios [27]. These architectural variations motivate our proposed enhancements that aim to combine the strengths of different approaches while mitigating their individual limitations.

Despite these architectural advantages, YOLOv12 exhibits specific limitations when applied to sesame field weed detection tasks, particularly in comparison to the architectural characteristics of its predecessors. First, the single-backbone architecture, while computationally efficient, constrains the network’s ability to simultaneously extract both fine-grained spatial features (essential for localizing small weed targets) and high-level semantic features (necessary for robust crop-weed discrimination under varying illumination). This limitation is shared across YOLOv5-v11, where single-backbone designs (CSPDarknet in YOLOv5, C2f-based in YOLOv8/v11) similarly prioritize inference speed over feature representation diversity. The inherent trade-off in single-backbone designs requires balancing spatial resolution preservation against semantic abstraction depth, which becomes problematic when processing agricultural imagery containing targets with extreme scale variation. Second, the C3K2 module in the backbone, designed for general object detection, exhibits potential parameter redundancy and limited cross-channel information exchange when processing agricultural imagery with subtle inter-class variations. This limitation is partially shared with YOLOv8’s C2f and YOLOv11’s enhanced backbone modules, which similarly employ standard convolution operations applying uniform receptive fields across all channels, lacking adaptability to the diverse feature requirements of morphologically similar crop and weed seedlings. Third, the standard FPN-based neck network employs static feature fusion through fixed element-wise addition, preventing adaptive adjustment of fusion weights based on scale-specific characteristics of input features. This design has remained largely unchanged across YOLOv5-v12 despite architectural innovations in backbone structures. This limitation is particularly relevant in agricultural scenarios where target scale distribution varies dramatically within individual images. These architectural constraints motivated the enhancements proposed in this study, as detailed in Section 2.2.

### 2.2. Improving the YOLOv12 Model Architecture

**Figure 1:**
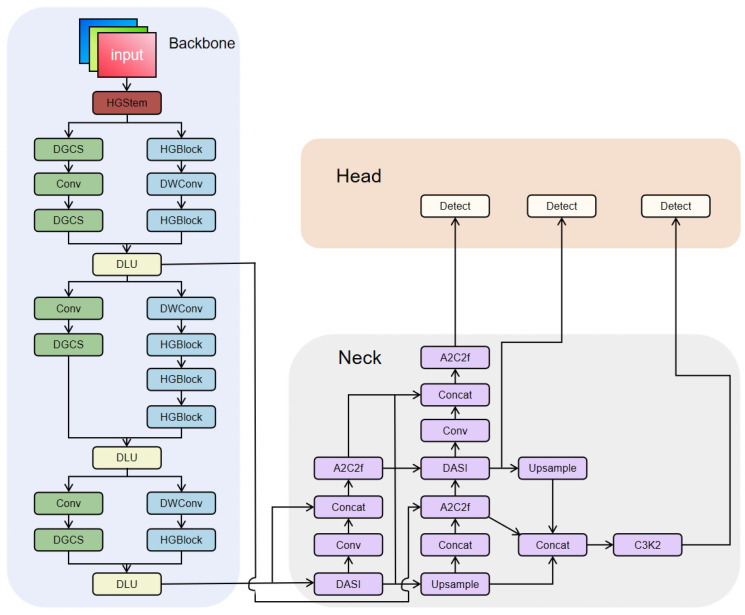
Improved YOLOv12-BDA Architecture.

The improved YOLOv12-BDA architecture is illustrated in Figure 1. First, we restructured the backbone from a single-backbone to a dual-backbone architecture by introducing HGStem and HGBlock, and we proposed a DLU module for deep feature extraction from images. This enhancement improved the model’s accuracy in detecting weeds. Second, replacing the C3K2 layer in the backbone network with the proposed DGCS module improves the model’s real-time performance for detecting small weed targets in complex agricultural backgrounds. Finally, integrating the DASI [31] module into the neck network addresses the issue of insufficient semantic information propagation in traditional feature pyramid networks, enabling the network to better capture detailed features of weed targets at different scales.

#### 2.2.1. Improvements to the Backbone Network (AFS Module)

The YOLOv12 backbone network exhibits limitations when processing complex sesame field scenarios, primarily manifested as insufficient feature extraction capabilities, inefficient multi-scale information fusion, and limited detection accuracy for small objects in densely planted environments. These limitations are particularly evident in sesame field scenarios where small weed targets often occupy a small fraction of the total image area and require highly discriminative feature representations to differentiate from visually similar sesame seedlings. Single-backbone architectures face an inherent design trade-off: shallow layers maintain high spatial resolution necessary for small object localization but lack semantic abstraction capability, while deep layers develop strong semantic features but lose critical spatial details through progressive downsampling. This architectural contradiction becomes problematic when the network must simultaneously handle targets with extreme scale variation—from small early-stage weed seedlings requiring preserved spatial detail to larger mature weeds benefiting from semantic robustness.

To address these issues, this study designed an Adaptive Feature Selection (AFS) dual-backbone architecture incorporating HGStem and HGBlock modules for complementary feature extraction pathways. The first backbone employs HGBlock modules with dense connections, prioritizing spatial detail preservation through multi-scale feature aggregation at shallow layers. This pathway maintains feature map resolution at 1/4 and 1/8 of input size across multiple processing stages, providing fine-grained spatial information essential for precise localization of small-targets. The second backbone utilizes efficient depthwise separable convolutions for semantic feature extraction with reduced computational overhead, focusing on high-level semantic abstraction necessary for robust classification. To mitigate excessive computational resource consumption on shallow feature maps, the dual-backbone design shares stem information, effectively reducing computational load and inference time.

A Dynamic Learning Unit (DLU) module is introduced to adaptively fuse features from the dual-backbone branches operating at the same spatial resolution. Note that the DLU module performs channel-level feature fusion without involving any upsampling or downsampling operations, as both input features and the output feature maintain identical spatial dimensions (H × W). By generating dynamic weights based on concatenated features from both backbones, the DLU module adaptively adjusts the contribution of each branch’s features, significantly enhancing the model’s feature representation capability and detection accuracy for weed detection in sesame fields. The mathematical formulation of the shared HGStem structure is defined as follows:

(1) $$F_{\mathrm{stem}1}=\mathrm{ReLU}\left({\mathrm{Conv}}_{3\times 3}^{s=2}\left(X_{0}\right)\right),$$

(2) $$F_{\mathrm{branch}1}={\mathrm{MaxPool}}_{2\times 2}^{s=1}\left(\mathrm{Pad}\left(F_{\mathrm{stem}1}\right)\right),$$

(3) $$F_{\mathrm{branch}2}=\mathrm{ReLU}\left({\mathrm{Conv}}_{2\times 2}^{s=1}\left(\mathrm{ReLU}\left({\mathrm{Conv}}_{2\times 2}^{s=1}\left(\mathrm{Pad}\left(F_{\mathrm{stem}1}\right)\right)\right)\right)\right),$$

(4) $$F_{\mathrm{stem}}=\mathrm{ReLU}\left({\mathrm{Conv}}_{1\times 1}\left(\mathrm{ReLU}\left({\mathrm{Conv}}_{3\times 3}^{s=2}\left(\mathrm{Concat}\left(F_{\mathrm{branch}1},F_{\mathrm{branch}2}\right)\right)\right)\right)\right),$$

The HGBlock module employs a densely connected architecture, achieving deep feature extraction through cascading multiple convolutional layers. Each HGBlock comprises n convolutional units, with the final output undergoing channel attention adjustment via a squeeze-encouraging mechanism. This design effectively enhances feature expressiveness while ensuring stable gradient propagation through residual connections.

(5) $$X_{i}={Conv}_{i}\left(X_{i-1}\right),i=1,2,\ldots ,n,$$

(6) $$F_{concat}=Concat\left(\left[X_{0},X_{1},X_{2},\ldots ,X_{n}\right],dim=1\right),$$

(7) $$F_{se}=EC\left(SC\left(F_{concat}\right)\right)$$

**Figure 2:**
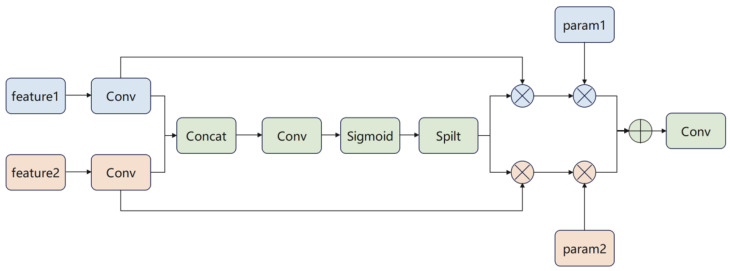
DLU Structure Diagram. The DLU module performs adaptive channel-level feature fusion between dual-backbone branches at the same spatial resolution, without changing feature map dimensions. The symbols ⊗ and ⊕ denote element-wise multiplication and element-wise addition, respectively.

The workflow of the DLU module comprises three stages: channel concatenation, dynamic weight generation, and adaptive fusion. Its structural diagram is shown in Figure 2. First, heterogeneous features (feature 1 and feature 2) from different branches undergo feature extraction through their respective convolutional layers (Conv). The extracted features are then concatenated (Concat) and merged into a unified feature set, ensuring consistency in feature dimensions. Subsequently, the features undergo further processing through a convolutional layer. A Sigmoid activation function generates dynamic alignment weights, which are then applied to weight the features. A Split operation divides the weighted features into two branches. These branches undergo fusion calculations with parameters param 1 and param 2, respectively. Finally, the results from both branches are fed into a convolutional layer for final feature integration and output.

The dynamic weight generation mechanism in the DLU module represents a critical design innovation for effective dual-backbone fusion. Unlike static fusion strategies (e.g., fixed-weight averaging or simple channel concatenation followed by 1 × 1 convolution) that treat features from both backbones uniformly regardless of input characteristics, the DLU module employs content-adaptive weighting to optimize fusion based on input image properties. The Sigmoid activation in the weight generation pathway ensures normalized weights in the range [0, 1], enabling smooth interpolation between the two feature streams. This design is motivated by the observation that different agricultural imaging conditions require different feature emphasis patterns. Images with complex backgrounds (dense soil textures, crop residues, varying shadow patterns) may benefit more from semantic features provided by the deeper backbone pathway, while images containing isolated small-targets against relatively uniform backgrounds may require stronger spatial localization features from the shallow pathway. The dynamic adaptation mechanism allows the network to automatically adjust fusion weights based on learned representations of input characteristics, improving robustness across diverse field conditions encountered in practical agricultural operations.

#### 2.2.2. DGCS Module

The C3K2 module exhibits limitations when processing agricultural imagery with subtle inter-class variations, including potential parameter redundancy and limited cross-channel information exchange. To address these challenges, we propose the Dynamic Grouped Convolution and Channel Mixing Transformer (DGCS) module that enhances feature representation while maintaining computational efficiency for small weed detection in complex agricultural backgrounds.

**Figure 3:**
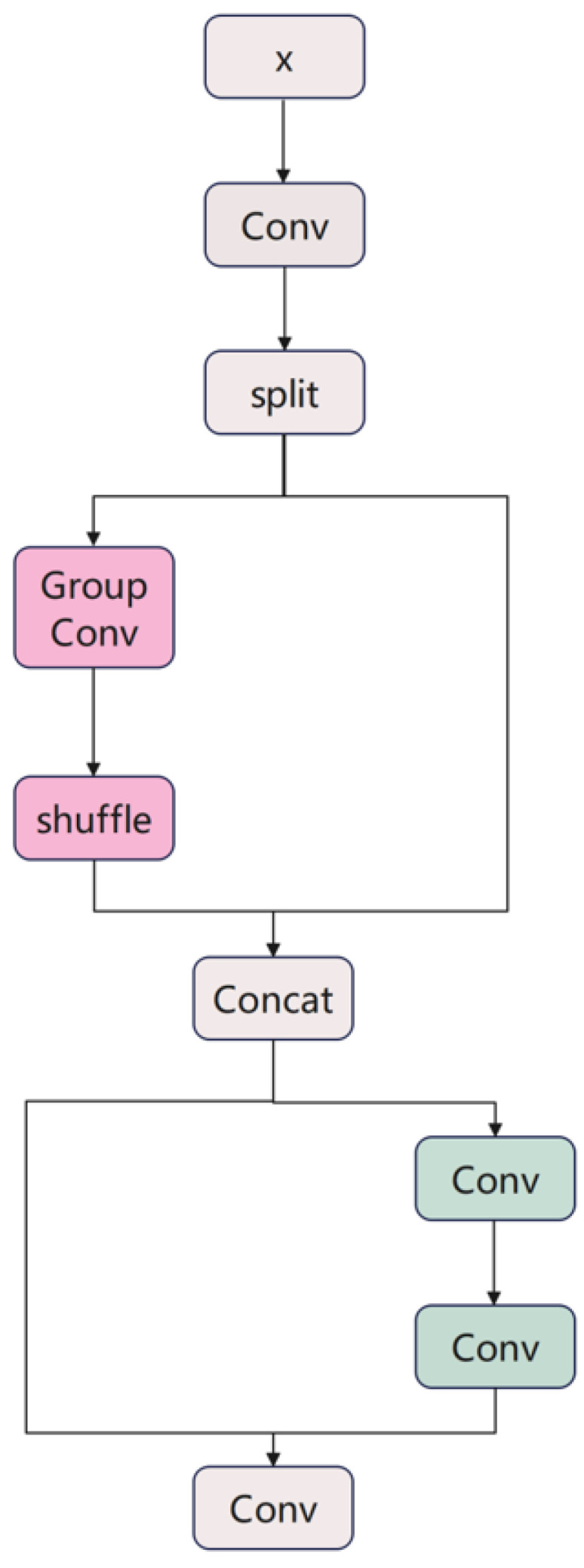
DGCS Structure Diagram.

The DGCS module achieves efficient feature learning through dynamic grouped convolution and channel mixing mechanisms. The workflow comprises four main stages: (1) Feature preprocessing—the input feature map X undergoes 1 × 1 convolution for channel-dimension transformation, followed by splitting into two sub-feature maps with a 1:3 ratio. (2) Dynamic grouped convolution—one sub-feature map (25% of channels) undergoes grouped convolution where the number of groups equals the channel count, achieving depthwise separable convolution. (3) Channel mixing transformation—a channel reshuffling operation redistributes features across groups to enable cross-group information exchange, followed by concatenation with the retained features. (4) Residual fusion—the concatenated features are fused with original branch features via residual connections, followed by refinement through two 1 × 1 convolutional layers. Figure 3 illustrates the DGCS module structure.

The channel reshuffling implements a systematic permutation: for input with C channels and G groups, channel c is mapped to position (c mod G) × (C/G) + floor (c/G), ensuring balanced redistribution where subsequent layers access mixed representations from all original groups.

#### 2.2.3. DASI Module

For sesame field weed detection tasks, traditional feature fusion strategies often fail to effectively handle semantic information differences between multi-scale targets. This causes small-scale weed targets to be easily overlooked, while large-scale crop features exhibit redundancy. To address this issue, this study adapts the Dimension-Aware Selective Integration (DASI) module from Zheng et al. [31] for infrared small object detection. By establishing a dynamic interaction mechanism among multi-level feature maps, this module achieves adaptive fusion and enhancement of features across different scales. The DASI module dynamically adjusts fusion weights based on the scale differences in input features. This effectively mitigates the semantic gap between high- and low-level features inherent in traditional FPN structures. Moreover, it enhances the network’s perception capabilities for multi-scale weed targets. This provides strong support for precise detection in complex agricultural environments.

**Figure 4:**
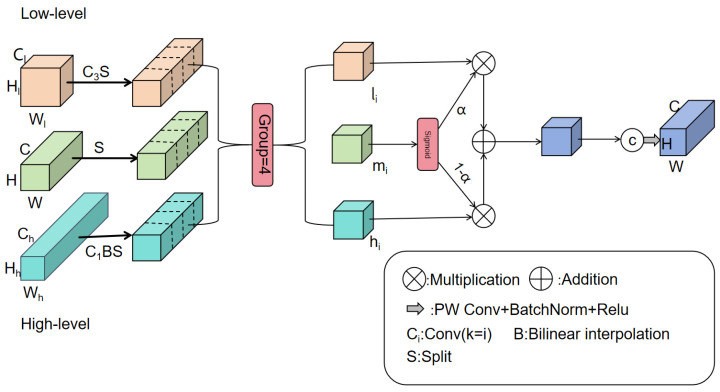
DASI Structure Diagram.

The workflow of the DASI module is as follows: it receives three feature maps of different scales (high-resolution features, current-resolution features, and low-resolution features) as input. Then, through scale alignment operations, it unifies the feature maps of different scales to the spatial dimensions of the current-resolution features. Subsequently, it is partitioned into four equal segments along the channel dimension, yielding ${\left(h_{i}\right)}_{i=1}^{4}\in$ $R^{H\times W\times \frac{C}{4}},{\left(l_{i}\right)}_{i=1}^{4}\in R^{H\times W\times \frac{C}{4}}$, and ${\left(m_{i}\right)}_{i=1}^{4}\in R^{H\times W\times \frac{C}{4}}$, where $h_{i}$, $l_{i}$, and $m_{i}$ represent the i-th partitioned feature of the high-resolution, low-resolution, and current-resolution features, respectively. The calculation formulas are given by Equations (8)–(10). Finally, the four fused sub-feature blocks are recombined via channel concatenation and undergo tail convolution, batch normalization, and activation function processing. The formula is shown in Equation (11), where $\alpha \in R^{H\times W\times \frac{C}{4}}$ represents the element-wise activation values obtained by applying the sigmoid function to $m_{i}$, Conv represents convolution, B denotes batch normalization, and δ indicates the rectified linear unit (ReLU). The element-wise weighting mechanism allows adaptive feature selection: spatial locations and channels with α>0.5 favor fine-grained features from $l_{i}$, while those with α<0.5 emphasize contextual features from $h_{i}$. The structural diagram is shown in Figure 4.

Traditional Feature Pyramid Networks (FPNs) employ static fusion strategies, typically using fixed element-wise addition (*F*_out = *F*_high + *F*_low) or concatenation followed by 1 × 1 convolution to combine features from adjacent pyramid levels. This approach operates under the implicit assumption that features from different scales contribute equally to detection performance across all input scenarios and all spatial locations. This assumption is problematic for agricultural weed detection where target scale distribution exhibits dramatic intra-image variation. Agricultural field images often simultaneously contain small weed seedlings that primarily benefit from fine-grained spatial detail in high-resolution features, and larger mature weeds that require semantic context from low-resolution features. Static fusion weights cannot adapt to this heterogeneous scale distribution, potentially resulting in suboptimal feature representations where different target sizes receive non-optimal feature combinations.

The DASI module addresses this limitation through scale-adaptive dynamic weighting, where fusion weights are computed based on activation characteristics of input feature maps rather than being predetermined. The core mechanism is defined in Equation (8): $\alpha =sigmoid\left(m_{i}\right)$, where $m_{i}$ represents the i-th channel partition of current-resolution features. The sigmoid function ensures $\alpha \in \left(0,1\right)$, enabling smooth interpolation between high-resolution ($h_{i}$) and low-resolution ($l_{i}$) features in Equation (9): $m_{i}^{\prime }=\alpha ⊙l_{i}+\left(1-\alpha \right)⊙h_{i}$. This formulation provides an interpretation: when current-resolution features exhibit high activation magnitudes ($m_{i}$ contains large positive values), sigmoid ($m_{i}$) approaches 1, increasing weight α and emphasizing low-resolution semantic features. Conversely, when current-resolution activations are relatively weak (small $m_{i}$ values), sigmoid ($m_{i}$) approaches 0, reducing α and prioritizing high-resolution spatial features. This mechanism allows the network to adapt fusion emphasis based on scale-dependent feature requirements inferred from current-resolution activation patterns.

The partition of features into four channel sub-blocks (Equations (9) and (10)) serves dual theoretical purposes. First, it enables fine-grained adaptive fusion at sub-feature granularity, allowing different channel groups to independently adjust their emphasis on different feature sources based on their semantic content. This design is conceptually motivated by the observation that different channel groups may encode different types of visual information (e.g., texture-sensitive channels vs. color-sensitive channels), which may benefit from different fusion strategies. Second, this partitioning reduces computational complexity: each sub-block requires only one sigmoid operation for weight generation, yielding a constant number (4) of weight computations per feature map regardless of total channel count C. This achieves O(1) computational complexity with respect to channel dimension, compared to O(C) for hypothetical per-channel dynamic weighting, making the approach scalable to high-dimensional feature representations typical in modern detection networks.

The theoretical foundation for dynamic weighting relates to the semantic gap problem in multi-scale object detection: shallow high-resolution features (e.g., P2 layer at 1/4 input resolution) contain precise edge localization and texture detail but lack semantic abstraction capability to distinguish objects from background clutter. Deep low-resolution features (e.g., P5 layer at 1/32 resolution) develop strong semantic representations but lose fine-grained spatial information through progressive pooling operations. By dynamically adjusting fusion weights based on target characteristics inferred from current-resolution activations, DASI is designed to bridge this semantic gap, theoretically enabling the network to leverage complementary strengths of features at different pyramid levels. This adaptive fusion strategy represents a departure from fixed-weight approaches in standard FPN architectures, potentially offering improved performance for datasets with heterogeneous multi-scale target distributions characteristic of agricultural field imagery.

(8) $$\alpha =sigmoid\left(m_{i}\right),$$

(9) $$m_{i}^{\prime }=\alpha ⊙l_{i}+\left(1-\alpha \right)⊙h_{i},$$

where ⊙ denotes element-wise multiplication. In Equation (8), the sigmoid function is applied element-wise to each spatial position and channel in $m_{i}$, producing dynamic fusion weights. Equation (9) performs adaptive element-wise fusion of high-resolution and low-resolution features based on these spatially varying weights.

(10) $$F_{u}^{\prime }=\left[m_{1}^{\prime },m_{2}^{\prime },m_{3}^{\prime },m_{4}^{\prime }\right],$$

(11) $$F_{u}^{\prime \prime }=\delta \left(B\left(Conv\left(F_{u}^{\prime }\right)\right)\right),$$

### 2.3. Dataset Description

This study utilized the publicly available “Crop and Weed Detection Data with Bounding Boxes” dataset, created by Panara Utsav, Pandya Raviraj, and Mohit Rayja in 2020 [32]. The dataset is accessible via the Kaggle platform at https://www.kaggle.com/datasets/ravirajsinh45/crop-and-weed-detection-data-with-bounding-boxes (accessed on 30 July 2025) and was specifically designed to support the development of precision weed management systems that minimize pesticide contamination risks while reducing agricultural chemical waste. The dataset comprises 1300 RGB images with standardized resolution of 512 × 512 pixels, capturing sesame (*Sesamum indicum* L.) plants and various weed species commonly encountered in sesame cultivation environments. Images represent realistic agricultural field conditions with varying plant densities, growth stages, and environmental backgrounds typical of operational farming scenarios.

All 1300 images contain bounding box annotations in YOLO format, where each annotation specifies class_id, x_center, y_center, width, and height with coordinates normalized to image dimensions. The dataset includes two object classes: sesame (crop) and weed. Each image contains multiple labeled instances, with an average of approximately three bounding box annotations per image, though individual images may contain significantly more instances depending on plant density and field conditions. The complete annotation coverage ensures comprehensive representation of both crop and weed targets across diverse spatial distributions and occlusion scenarios. The dataset encompasses natural agricultural variability including diverse soil backgrounds such as bare soil, crop residue, and varying moisture levels. Images were collected under different lighting conditions characteristic of field data collection, incorporating realistic challenges including partial occlusion between plants, overlapping foliage, shadow variations, and mixed maturity stages of both crops and weeds. This variability reflects practical deployment conditions for weed detection systems in commercial agricultural operations.

Following standard practice for deep learning model development, the complete dataset of 1300 images was randomly partitioned into training, validation, and test subsets using an 8:1:1 ratio. This division yielded 1040 images (80%) for model training, 130 images (10%) for validation during hyperparameter tuning, and 130 images (10%) for final performance evaluation. The random partitioning strategy ensures balanced representation of imaging conditions, object densities, and environmental variations across all three subsets, supporting robust model training and unbiased generalization assessment.

#### 2.3.1. Data Augmentation Strategy

To enhance model robustness across diverse field conditions, online data augmentation was applied to the training set during model training. The augmentation pipeline comprises three categories of transformations designed to address specific challenges in agricultural weed detection.

Color space augmentations simulate natural illumination variations through HSV (Hue-Saturation-Value) color space adjustments. Hue shifting (±1.5%) accounts for color cast variations caused by different solar angles and atmospheric conditions. Saturation modification (±70%) simulates changes in lighting intensity and sensor response characteristics. Brightness adjustment (±40%) reproduces the effects of cloud cover transitions and shadow patterns commonly encountered during field data collection.

Geometric transformations address scale and spatial variations inherent in agricultural imaging scenarios. Spatial translation (±10%) simulates camera positioning variability and target displacement within the field of view. Multi-scale training with scale factor 0.5 enables processing at 0.5× to 1.5× of the original image size, improving detection robustness across different imaging distances and target sizes. Horizontal flipping (50% probability) augments training samples while maintaining semantic validity, as weed orientation exhibits horizontal symmetry. Mosaic augmentation combines four randomly selected images into a single training sample, enhancing the model’s ability to detect small-targets by increasing target density per training iteration [33]. This augmentation is disabled during the final 10 epochs to stabilize training convergence.

Occlusion-based augmentations simulate partial target visibility conditions caused by overlapping foliage and inter-plant occlusion. RandAugment [34] applies a randomly selected sequence of augmentation operations with automated magnitude tuning, reducing the need for manual hyperparameter optimization. Random erasing [35] randomly masks rectangular regions (40% application probability) to simulate occlusion patterns and improve robustness to partial target visibility.

The complete augmentation configuration is presented in Table 1. All transformations are applied stochastically during training using the PyTorch framework’s built-in augmentation pipeline. Additional geometric transformations (rotation, shear, perspective distortion, vertical flipping) and advanced composition methods (BGR channel swapping, mixup blending) were disabled to prevent excessive distortion that may compromise small-target feature integrity in agricultural imagery.

#### 2.3.2. WeedCrop Image Dataset

To evaluate the generalization capability of the proposed YOLOv12-BDA architecture, cross-dataset validation was conducted using the publicly available WeedCrop Image Dataset [36]. The WeedCrop dataset, created by Sudars et al. [36] in 2020, comprises 2822 RGB images with bounding box annotations in YOLO v5 PyTorch format. Images were captured using three camera systems (Intel RealSense D435, Canon EOS 800D, and Sony W800) under both controlled greenhouse environments and field conditions. The dataset contains 6 food crop species and 8 weed species, with 7853 total annotations across two classes: “Crop” and “Weed.”

The WeedCrop dataset differs from the sesame dataset in several key aspects: (1) imaging hardware—multiple camera systems with varying sensor specifications versus single-camera acquisition; (2) species composition—completely different crop and weed species with no overlap; (3) environmental conditions—combination of controlled greenhouse and outdoor field settings versus consistent field conditions; and (4) growth stages—deliberate inclusion of diverse developmental stages from seedlings to mature plants. These substantial differences constitute a significant domain gap that makes the WeedCrop dataset suitable for evaluating cross-domain transfer capability rather than within-domain generalization. The evaluation assesses whether the architectural improvements designed for sesame weed detection demonstrate robustness when transferred to different crop-weed detection tasks, thereby testing the fundamental soundness of the proposed architectural design. The WeedCrop dataset was partitioned using an 8:1:1 split, yielding 2257 training images, 282 validation images, and 283 test images.

### 2.4. Test Environment

The experimental platform for this study is Windows 11, featuring an AMD Ryzen 7 7735 H CPU with Radeon Graphics (AMD Inc., Santa Clara, CA, USA) and an NVIDIA GeForce RTX 4060 GPU (NVIDIA Corporation, Santa Clara, CA, USA). The programming language used is Python version 3.9.21, and the deep learning framework is PyTorch version 2.5.1. The training was configured for 300 epochs based on preliminary convergence experiments. Due to GPU memory constraints (NVIDIA GeForce RTX 4060 with 8 GB VRAM), the batch size was set to 4. Following the official YOLOv12 implementation [27], we adopted the recommended hyperparameters: momentum factor of 0.937, weight decay of 0.0005, and initial learning rate of 0.01 with cosine annealing scheduler. To ensure fair comparison across all baseline models (YOLOv5n, YOLOv8n, YOLOv10n, YOLOv11n, and YOLOv12n), identical hyperparameter configurations were maintained throughout all experiments.

### 2.5. Evaluation Indicators

To evaluate the YOLOv12-BDA model for detecting weeds in sesame fields, this study employs precision, recall, and average precision metrics (mAP@50 and mAP@50:95). Their calculation formulas are presented in Equations (12)–(15).

(12) $$\mathrm{Precision}=\frac{TP}{TP+FP}$$

(13) $$\mathrm{Recall}=\frac{TP}{TP+FN}$$

(14) $$AP=\int_{0}^{1}P\left(R\right)dR$$

(15) $$mAP=\frac{1}{N}\sum_{i=1}^{N}{AP}_{i}$$

Among these, TP denotes the number of samples correctly predicted as positive by the model (true positives), FP denotes the number of samples incorrectly predicted as positive by the model (false positives), FN denotes the number of samples incorrectly predicted as negative by the model (false negatives), $P\left(R\right)$ denotes precision as a function of recall R, AP denotes the area under the Precision-Recall curve for a single category (computed as the integral of precision over recall from 0 to 1), and N denotes the total number of classification categories in the dataset.

## 3. Results

### 3.1. Analysis of Weed Identification Results in Sesame Fields

To validate the effectiveness of the improved model for detecting weeds in sesame fields, comprehensive comparison experiments were conducted against representative baseline architectures spanning different detection paradigms. The baseline selection encompasses two categories: (1) YOLO-series models (YOLOv5n through YOLOv12n) representing the evolutionary trajectory of single-stage detectors with progressive architectural innovations—from anchor-based detection (YOLOv5n) to anchor-free paradigms (YOLOv8n), decoupled prediction heads (YOLOv10n), enhanced feature extraction (YOLOv11n), and attention-centric design (YOLOv12n); and (2) RT-DETR, a transformer-based detector employing encoder–decoder architecture with deformable attention mechanisms, representing fundamentally different detection philosophy from CNN-centric YOLO frameworks. This selection enables systematic evaluation across architectural generations and design paradigms while maintaining experimental consistency. Additional architectural diversity is provided through backbone comparison experiments (Section 3.2.1) evaluating efficiency-optimized networks (FasterNet, StarNet), compound scaling strategies (EfficientNet-B0), and hybrid CNN-Transformer architectures (EfficientViT). The performance of all compared models was evaluated on the same dataset, with experimental results presented in Table 2.

As shown in Table 2, YOLOv12n achieves precision of 86.95% and mAP@50 of 86.90%, demonstrating competitive performance among baseline models. However, its mAP@50:95 of 51.37% indicates room for improvement in precise localization. YOLOv12n was selected as the baseline for optimization due to its balanced performance and attention-centric architectural foundation suitable for small-target detection enhancement.

Compared to YOLOv12n, YOLOv12-BDA achieves substantial improvements: mAP@50 of 91.57% (+4.67%), mAP@50:95 of 67.46% (+16.09%), and recall of 87.60% (+3.29%). While precision decreases slightly to 85.36% (−1.59%), the model maintains high accuracy while significantly improving detection completeness. These results demonstrate the effectiveness of the proposed architectural modifications in addressing small-target detection challenges in agricultural scenarios.

Complexity analysis reveals that YOLOv12-BDA requires 4.51 M parameters and 10.7 GFLOPs, representing increases of 76.2% and 69.8% over baseline YOLOv12n (2.56 M parameters, 6.3 GFLOPs). These increases reflect the computational cost of dual-backbone architecture and dynamic fusion mechanisms. However, the 16.09% improvement in mAP@50:95—substantially exceeding the 4.67% gain in mAP@50—demonstrates significant enhancement in localization precision, which is critical for practical deployment. The performance-complexity relationship yields 0.21% mAP@50:95 gain per 1% parameter increase, indicating a design trade-off prioritizing detection accuracy over computational efficiency.

Inference efficiency analysis demonstrates that YOLOv12-BDA maintains real-time processing capability despite increased computational requirements. The proposed model achieves an inference time of 8.840 ms (24.8% increase over YOLOv12n’s 7.085 ms), processing approximately 113 frames per second—substantially exceeding agricultural robotic platform requirements (≥30 FPS). Baseline models demonstrate inference times ranging from 4.771 ms (YOLOv5n) to 7.085 ms (YOLOv12n), while RT-DETR requires 12.027 ms. The performance-efficiency ratio shows YOLOv12-BDA achieves 10.35% mAP@50 per ms and 7.63% mAP@50:95 per ms, compared to 12.26% and 7.25% for YOLOv12n. The 16.09% mAP@50:95 improvement—substantially exceeding the 4.67% mAP@50 gain—demonstrates enhanced localization precision that justifies the computational overhead for applications prioritizing detection quality.

**Figure 5:**
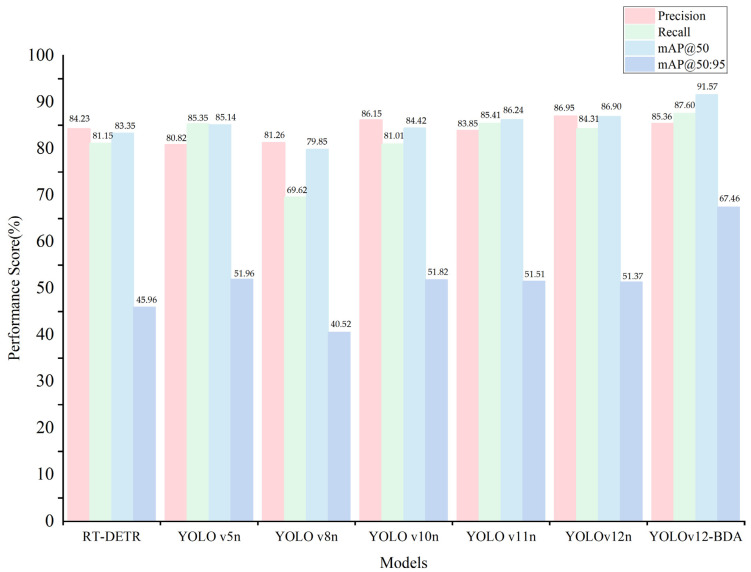
Performance comparison of evaluated YOLO models across multiple metrics.

Figure 5 presents the performance comparison of all evaluated models across four key metrics: Precision, Recall, mAP@50, and mAP@50:95. YOLOv12-BDA achieves the highest mAP@50 (91.57%) and mAP@50:95 (67.46%), representing improvements of 4.67% and 16.09% over baseline YOLOv12n, respectively, while maintaining balanced precision (85.36%) and recall (87.60%). The grouped bar chart format ensures clear visibility of all model performances, facilitating accurate comparison across methods.

**Figure 6:**
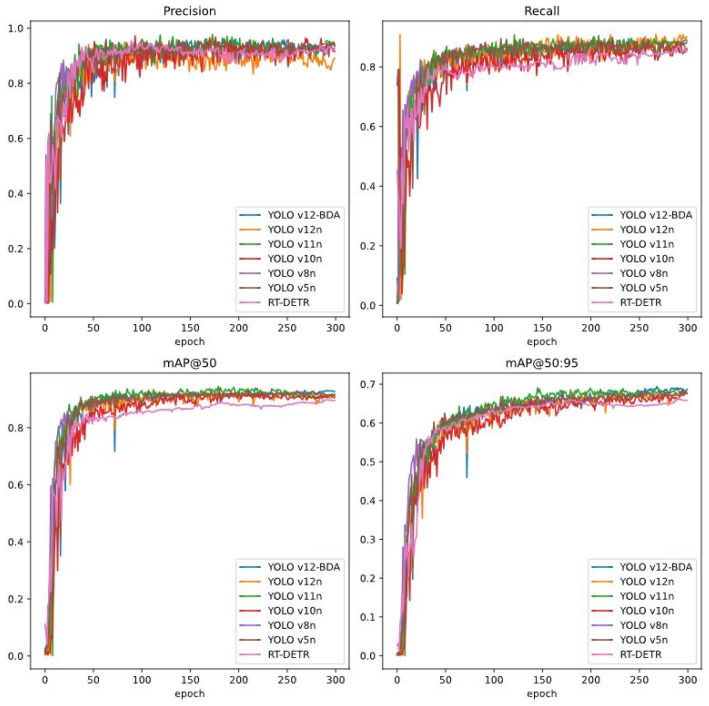
Training convergence curves of evaluated YOLO models showing the evolution of Precision, Recall, mAP@50, and mAP@50:95 over 300 epochs on the sesame weed detection dataset.

Figure 6 presents training convergence curves for all evaluated models. All models exhibit rapid convergence during the initial 100 epochs and stabilize by epoch 250–300. YOLOv12-BDA demonstrates faster initial convergence, maintains consistently superior final performance (Precision: 85.36%, Recall: 87.60%, mAP@50: 91.57%, mAP@50:95: 67.46%), and exhibits smoother convergence trajectories. These characteristics confirm that 300 epochs provide sufficient training duration and validate the appropriateness of the training configuration.

### 3.2. Comparative Experiments on Architecture Components

#### 3.2.1. Backbone Network Comparison

The backbone network serves as the core feature extraction component, with its design directly impacting feature representation capabilities and computational efficiency. To validate the performance of the proposed AFS dual-backbone architecture, comparative experiments were conducted with representative lightweight backbones: EfficientNet-B0 (compound scaling with MBConv), FasterNet (partial convolution), StarNet (star-shaped topology), and EfficientViT (CNN-Transformer hybrid). The experimental results are shown in Table 3.

As shown in Table 3, YOLOv12-AFS achieves the highest performance with mAP@50 of 89.96% and mAP@50:95 of 51.69%, outperforming all alternative backbones. Compared to YOLOv12-timm (EfficientNet-B0), YOLOv12-AFS demonstrates 4.49% higher mAP@50 and 1.82% higher mAP@50:95, suggesting that compound scaling strategies optimized for ImageNet classification may not transfer optimally to agricultural small-target detection. YOLOv12-FasterNet achieves competitive precision (85.55%) but remains 3.41% lower in mAP@50 than YOLOv12-AFS, potentially indicating that aggressive channel reduction may discard discriminative features essential for distinguishing morphologically similar crop and weed foliage. YOLOv12-StarNet exhibits the lowest precision (74.81%), suggesting that centralized aggregation patterns may create information bottlenecks. YOLOv12-EfficientViT achieves moderate performance (mAP@50: 87.12%) while requiring substantial computational overhead, indicating that Transformer components provide limited advantage for agricultural imagery where discriminative features remain predominantly local.

Computational complexity and efficiency analysis reveals significant variations among backbone architectures. YOLOv12-StarNet achieves the lowest complexity with 1.54 M parameters and 4.5 GFLOPs but exhibits substantially lower precision (74.81%). YOLOv12-FasterNet demonstrates balanced efficiency with 3.41 M parameters and 8.6 GFLOPs, achieving competitive precision (85.55%). In contrast, YOLOv12-EfficientViT exhibits the highest requirements with 12.44 M parameters and 33.0 GFLOPs (173% more than YOLOv12-AFS) but achieves only moderate performance improvement (mAP@50: 87.12%). YOLOv12-AFS achieves optimal performance-complexity balance with 4.55 M parameters and 9.4 GFLOPs, delivering the highest accuracy with moderate computational requirements. The efficiency ratio analysis shows that YOLOv12-AFS achieves 19.77% mAP@50 per million parameters, compared to 14.06% for YOLOv12-timm and 18.99% for YOLOv12-FasterNet.

Inference efficiency analysis demonstrates distinct performance characteristics across backbone architectures. YOLOv12-StarNet achieves the fastest inference at 5.534 ms, followed by YOLOv12-AFS at 7.046 ms and YOLOv12-FasterNet at 7.561 ms. However, StarNet’s 21.5% faster inference comes at the cost of substantially degraded accuracy (mAP@50: 86.33% vs. 89.96%). YOLOv12-timm requires 8.038 ms (14.1% slower than AFS), while YOLOv12-EfficientViT exhibits the longest inference time of 11.372 ms (61.4% slower than AFS), reflecting the computational overhead of vision transformer components. The performance-efficiency ratio shows that YOLOv12-AFS achieves 12.76% mAP@50 per ms inference time, substantially outperforming YOLOv12-timm (10.64% per ms), YOLOv12-FasterNet (11.45% per ms), YOLOv12-StarNet (15.60% per ms with poor accuracy), and YOLOv12-EfficientViT (7.66% per ms). These results confirm that the proposed dual-backbone AFS architecture achieves superior accuracy-efficiency balance, delivering the highest detection performance with competitive inference speed suitable for real-time agricultural applications.

#### 3.2.2. Upsampling Modules Comparison

Feature pyramid networks require upsampling operations to align multi-scale features during the fusion process. The choice of upsampling method significantly impacts detection accuracy, particularly for small-target identification in agricultural scenarios where precise spatial detail preservation is critical. To validate the upsampling strategy adopted in the DASI module (Section 2.2.3), we conducted comparative experiments with several state-of-the-art upsampling methods.

The comparison includes three representative upsampling approaches: DySample [37], CARAFE [38], and EUCB [39]. DySample employs dynamic point sampling with offset prediction to generate content-aware upsampling kernels, enabling adaptive interpolation based on local feature characteristics. CARAFE (Content-Aware ReAssembly of FEatures) predicts pixel-specific reassembly kernels through lightweight sub-networks, allowing fine-grained control over the feature aggregation process during upsampling. EUCB (Efficient Upsampling with Convolutional Blocks) utilizes traditional upsampling operations combined with depth-wise separable convolutions for efficient feature upsampling. Each method represents a distinct design philosophy for addressing the spatial resolution alignment challenge in multi-scale feature fusion.

The experimental configuration maintains consistency across all compared methods. All models are based on the YOLOv12n architecture, with only the upsampling module in the neck network being varied. The training protocol follows identical hyperparameter settings: 300 epochs, batch size of 4, initial learning rate of 0.01 with cosine annealing, momentum factor of 0.937, and weight decay of 0.0005. All experiments are conducted on the same sesame weed detection dataset described in Section 2.3, using the standard 8:1:1 train-validation-test split.

The experimental results are presented in Table 4. YOLO12-DASI achieves the highest performance across all evaluation metrics, with mAP@50 of 89.59%, mAP@50:95 of 61.37%, precision of 89.40%, and recall of 88.74%. These results represent substantial improvements of 1.22%, 8.95%, 0.78%, and 0.80% in mAP@50, mAP@50:95, precision, and recall, respectively, compared to the second-best method (YOLO12-EUCB: 88.37% mAP@50, 52.42% mAP@50:95).

Among the alternative upsampling methods, DySample demonstrates competitive mAP@50 performance (87.75%) but exhibits limited improvement in mAP@50:95 (50.73%), suggesting potential limitations in precise localization at stricter IoU thresholds. The dynamic sampling mechanism in DySample requires additional computational overhead for offset prediction, yet this increased complexity does not translate to proportional accuracy gains for agricultural small-target detection. CARAFE achieves moderate mAP@50 (85.55%) with the longest inference time (12.57 ms), indicating substantial computational costs from its pixel-specific kernel prediction mechanism. While CARAFE’s content-aware reassembly strategy shows promise for general object detection, the computational overhead may limit its deployment feasibility on resource-constrained agricultural platforms. EUCB demonstrates balanced performance with 88.37% mAP@50 and 52.42% mAP@50:95, representing the second-best method in the comparison. The efficiency of EUCB stems from its use of depth-wise separable convolutions instead of standard convolutions, achieving computational savings while maintaining feature enhancement capability. However, its performance remains inferior to DASI across all metrics.

The superior performance of YOLO12-DASI can be attributed to its dynamic adaptive weighting mechanism that adjusts fusion weights based on feature activation patterns rather than employing fixed upsampling strategies. Unlike conventional upsampling methods that apply uniform operations across all spatial locations, DASI adaptively emphasizes different feature sources (high-resolution vs. low-resolution) based on local content characteristics inferred from current-resolution activations. This content-adaptive fusion strategy proves particularly effective for agricultural weed detection scenarios characterized by extreme intra-image scale variation, where small early-stage weed seedlings and large mature plants coexist within individual images and require different feature emphasis patterns.

From a computational efficiency perspective, YOLO12-DASI maintains competitive inference time (9.42 ms) despite incorporating dynamic weighting mechanisms, representing only modest overhead compared to simpler methods like DySample (8.63 ms) and EUCB (8.79 ms). The parameter count (2.62 M) and computational complexity (7.4 GFLOPs) represent increases of 2.34% and 17.46%, respectively, compared to baseline YOLOv12n (2.56 M parameters, 6.3 GFLOPs). Notably, YOLO12-DASI achieves these improvements with identical parameter count to YOLO12-EUCB (both 2.62 M), demonstrating that the performance advantage stems from architectural design rather than increased model capacity. The moderate computational overhead (7.4 GFLOPs vs. 6.7 GFLOPs for EUCB) yields substantial accuracy gains, with mAP@50:95 improving from 52.42% to 61.37%—an 8.95% increase. This efficiency-accuracy balance makes DASI particularly suitable for deployment in precision agriculture applications where both detection performance and real-time processing capabilities are essential.

These experimental results validate the effectiveness of the DASI module design for multi-scale feature fusion in agricultural weed detection. The dynamic adaptive weighting mechanism demonstrates clear advantages over both learning-based upsampling methods (DySample, CARAFE, EUCB) and conventional fixed interpolation strategies. The substantial performance improvement (8.95% gain in mAP@50:95 over EUCB) confirms that content-adaptive fusion strategies tailored to the specific characteristics of agricultural imagery—extreme scale variation, morphological similarity between crops and weeds, complex backgrounds—offer superior detection capabilities compared to general-purpose upsampling methods optimized for natural image datasets.

### 3.3. Ablation Experiments

To validate the effectiveness of the three proposed modules, ablation experiments were conducted using YOLOv12n as the baseline. Eight experimental configurations were evaluated, with results presented in Table 5.

As shown in Table 5, the experiments evaluate individual modules (Groups 2–4), pairwise combinations (Groups 5–7), and full integration (Group 8). The baseline YOLOv12n (Group 1) achieves mAP@50 of 86.90% and mAP@50:95 of 51.37%. Among individual modifications, the DASI module (Group 4) demonstrates the most significant improvement, with mAP@50 increasing to 89.59% (+2.69%) and mAP@50:95 to 61.37% (+10.00%). The AFS module (Group 2) yields notable gains in mAP@50 (+3.06%) and recall (+3.60%) but minimal improvement in mAP@50:95 (+0.32%). The DGCS module alone (Group 3) shows limited effectiveness, with all metrics declining, indicating that this modification requires complementary enhancements.

Pairwise combinations reveal synergistic effects. Group 5 (AFS + DGCS) achieves mAP@50 of 90.52% (+3.62%) with balanced precision (89.81%) and recall (86.08%). Group 6 (AFS + DASI) demonstrates substantial improvements in mAP@50 (+3.51%) and mAP@50:95 (+7.65%). Group 7 (DGCS + DASI) outperforms standalone DGCS, confirming the benefit of module integration. The complete integration (Group 8) achieves the highest performance with mAP@50 of 91.57% (+4.67%) and mAP@50:95 of 67.46% (+16.09%), demonstrating strong complementarity among the proposed modifications.

Inference efficiency analysis reveals distinct computational characteristics across module combinations. The baseline YOLOv12n achieves 7.085 ms inference time. Individual module additions exhibit varying impacts: AFS maintains comparable speed at 7.046 ms (0.6% improvement), DGCS reduces inference time to 6.853 ms (3.3% improvement) through parameter-efficient design, while DASI increases inference time to 9.422 ms (33.0% increase) due to dynamic multi-scale feature weighting. Pairwise combinations show synergistic efficiency patterns. Group 5 (AFS + DGCS) achieves 7.194 ms (1.5% increase), demonstrating that DGCS efficiency partially offsets AFS overhead. Group 6 (AFS + DASI) requires the longest inference time of 10.156 ms (43.3% increase), while Group 7 (DGCS + DASI) achieves 5.493 ms (22.5% reduction), benefiting from DGCS parameter efficiency. The final Group 8 configuration achieves 8.840 ms (24.8% increase), maintaining real-time capability at approximately 113 FPS while delivering the highest accuracy (mAP@50:95: 67.46%). The performance-efficiency ratio shows that Group 8 achieves 7.63% mAP@50:95 per ms, substantially outperforming baseline (7.25% per ms), confirming that the accuracy improvements (16.09% gain in mAP@50:95) justify the moderate inference overhead.

Individual module contribution analysis highlights the DASI module’s critical role. Among modules tested independently, DASI (Group 4) achieves the largest improvement with mAP@50:95 increasing by 10.00%, representing the most substantial single-module contribution to performance enhancement. This highlights DASI’s effectiveness in addressing multi-scale detection challenges in agricultural scenarios. The synergistic combination of all three modules (Group 8) achieves cumulative improvements of 4.67% in mAP@50 and 16.09% in mAP@50:95, demonstrating complementary functionality across the proposed architectural innovations.

**Figure 7:**
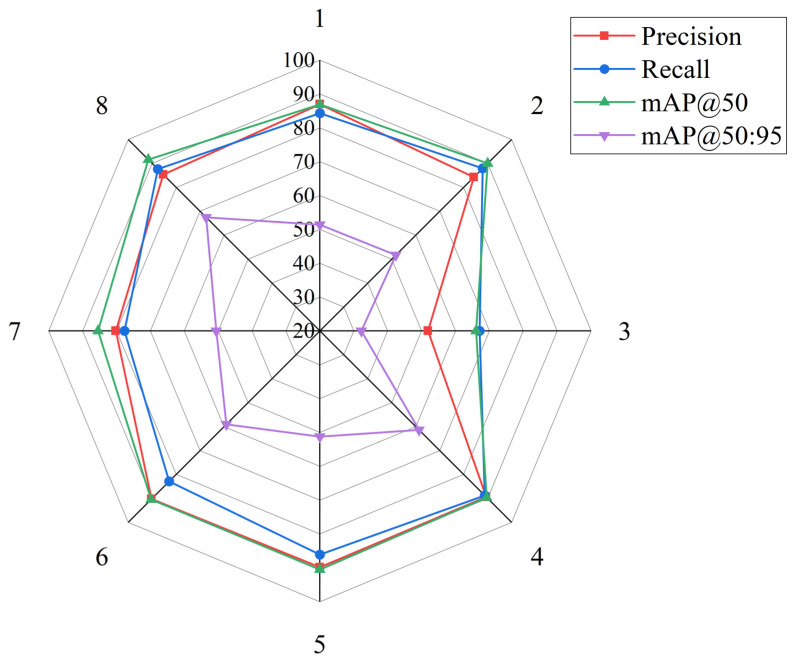
Radar chart of detection performance metrics for YOLOv12-BDA and baseline YOLO models.

Figure 7 presents a radar chart visualizing performance across experimental configurations. The precision metric maintains values above 80% for seven of eight groups, with Group 3 showing notable decline (51.88%) due to standalone DGCS implementation. The recall metric demonstrates typical precision-recall trade-offs, with higher recall values at Groups 2 and 8. The mAP@50 metric achieves strong performance (85–92%) for most configurations, with Group 8 reaching the highest value of 91.57%. The mAP@50:95 metric forms a smaller inner contour due to stricter localization requirements, ranging from 32.23% to 67.46%. Group 8 achieves the highest values across all metrics, confirming the effectiveness of combining all proposed improvements.

### 3.4. Hyperparameter Sensitivity Analysis

To validate the robustness of the proposed YOLOv12-BDA architecture across different training configurations, we conducted a systematic learning rate sensitivity analysis. Learning rate represents one of the most critical hyperparameters governing model convergence behavior and final detection performance. This analysis evaluates how variations in learning rate affect the model’s detection accuracy while maintaining all other hyperparameters constant.

We evaluated five learning rate values: 0.001, 0.01, 0.015, 0.02, and 0.1. All experiments maintained identical configurations for other hyperparameters (momentum factor of 0.937, weight decay of 0.0005, batch size of 4, and 300 training epochs). The experimental results are presented in Table 6.

As shown in Table 6, YOLOv12-BDA achieves optimal performance at the learning rate of 0.01, with mAP@50 of 91.57% and mAP@50:95 of 67.46%. This optimal setting yields balanced performance across both precision (85.36%) and recall (87.60%). The consistency between this optimal learning rate and the default configuration employed in our comparative experiments (Table 2, Table 3 and Table 4) confirms the appropriateness of our hyperparameter selection.

At the low learning rate of 0.001, the model exhibits performance degradation with mAP@50 decreasing to 85.67% and mAP@50:95 to 52.97%, representing reductions of 5.90% and 14.49%, respectively. The relatively modest decline in mAP@50 compared to the substantial reduction in mAP@50:95 indicates that while the model maintains reasonable localization capability at coarse IoU thresholds, it struggles to achieve precise bounding box regression required for stricter evaluation criteria.

Increasing the learning rate to 0.015 results in performance degradation, with mAP@50 declining to 85.74% and mAP@50:95 to 56.22%. At the learning rate of 0.02, a notable precision-recall trade-off emerges: precision increases to 88.29% (+2.93% compared to optimal), while recall decreases significantly to 77.28% (−10.32%). This asymmetric pattern suggests that higher learning rates favor confident predictions of clearly identifiable targets while causing the model to miss more challenging instances.

At the high learning rate of 0.1, the model maintains relatively stable performance with mAP@50 of 88.52% and mAP@50:95 of 56.90%. However, both precision (85.31%) and recall (81.59%) remain below the optimal configuration, indicating suboptimal feature learning despite avoiding complete training collapse.

The sensitivity analysis reveals that YOLOv12-BDA demonstrates reasonable robustness to learning rate variation, maintaining functional performance across a wide range of settings (0.001 to 0.1). The model achieves optimal performance at the default learning rate configuration (0.01), with consistent performance patterns characterized by gradual improvement from low learning rates to the optimal point, followed by progressive degradation at higher values. These results confirm the stability of the proposed architecture across different training configurations.

### 3.5. Cross-Domain Transfer Evaluation

To evaluate the cross-domain transferability of the proposed architectural improvements, experiments were conducted on the WeedCrop Image Dataset. As described in Section 2.3.2, the substantial differences between datasets in species composition, imaging systems, and environmental conditions constitute a significant domain gap, making this evaluation a cross-domain transfer assessment rather than conventional within-domain generalization testing. Table 7 presents the comparative performance results of all evaluated YOLO architectures on the WeedCrop dataset.

As shown in Table 7, all models demonstrate substantial performance degradation on the WeedCrop dataset compared to the sesame dataset (Table 2), with average reductions of approximately 15–20% in mAP@50 and 15–18% in mAP@50:95 across baseline models. This significant performance drop quantitatively reflects the large domain gap between the two datasets, encompassing different crop and weed species, multiple camera systems with varying sensor specifications, and mixed environmental conditions (controlled greenhouse versus field settings). The magnitude of this performance reduction is characteristic of cross-domain transfer scenarios where models must adapt to fundamentally different visual feature distributions.

YOLOv12-BDA achieves mAP@50 of 79.65% and mAP@50:95 of 45.95%, representing improvements of 6.44% and 9.11% over baseline YOLOv12n, respectively. These performance gains are comparable in magnitude to those observed on the sesame dataset (4.67% improvement in mAP@50, 16.09% improvement in mAP@50:95 from Table 2), demonstrating consistent benefits across different agricultural scenarios. The recall metric shows particularly notable improvement, with YOLOv12-BDA achieving 77.85% compared to YOLOv12n’s 73.41% (4.44% improvement), while maintaining competitive precision at 72.87%.

Comparing baseline architectures reveals differential cross-domain transfer capabilities. YOLOv5n exhibits the largest performance degradation (mAP@50: 85.14% on sesame→61.08% on WeedCrop), suggesting greater sensitivity to domain shift. YOLOv12n demonstrates relatively better transfer performance (73.21% mAP@50 on WeedCrop), while YOLOv12-BDA’s superior performance (79.65% mAP@50) indicates that the proposed dual-backbone architecture with dynamic feature fusion provides additional robustness under domain shift.

Cross-domain complexity analysis demonstrates consistent performance-efficiency trade-off characteristics. On the WeedCrop dataset, YOLOv12-BDA requires 4.51 M parameters and 10.7 GFLOPs, representing increases of 79.7% in parameters (from 2.51 M to 4.51 M) and 84.5% in GFLOPs (from 5.8 to 10.7) compared to YOLOv12n (2.51 M parameters, 5.8 GFLOPs). The inference time increases from 8.095 ms (YOLOv12n) to 10.532 ms (YOLOv12-BDA), representing a 30.1% overhead that maintains real-time capability for agricultural deployment scenarios. These computational increases correspond to 6.44% mAP@50 and 9.11% mAP@50:95 improvements, yielding efficiency ratios of 0.081% mAP@50 gain per 1% parameter increase and 0.11% mAP@50:95 gain per 1% parameter increase. Compared to YOLOv5n (2.18 M parameters, 5.8 GFLOPs, 61.08% mAP@50), YOLOv12-BDA achieves 18.57% mAP@50 improvement with 2.33 M additional parameters. The comparable performance-complexity relationships observed on WeedCrop (0.081% mAP@50 per 1% parameter increase) and the sesame dataset (0.21% mAP@50:95 per 1% parameter increase) indicate that the architectural modifications maintain stable efficiency-accuracy characteristics under cross-domain transfer, though the absolute performance reduction highlights the need for domain-specific adaptation in practical deployment scenarios.

Despite the substantial domain gap and resulting performance degradation, YOLOv12-BDA maintains relative performance advantages over baseline YOLOv12n across both datasets, achieving improvements of 6.44% and 9.11% in mAP@50 and mAP@50:95 on WeedCrop compared to 4.67% and 16.09% on sesame. This preservation of relative improvement under cross-domain transfer suggests that the proposed architectural modifications—the AFS dual-backbone with DLU module, DGCS module, and DASI module—provide architectural robustness that extends beyond the specific sesame weed detection task. However, the substantial absolute performance reduction (mAP@50:95 from 67.46% to 45.95%, a 21.51 percentage point decrease) indicates that direct deployment to new crop systems without domain-specific adaptation requires careful validation. The cross-domain transfer results demonstrate architectural soundness but should not be interpreted as evidence of broad agricultural generalization without further systematic evaluation across diverse crop types, geographical regions, and environmental conditions.

### 3.6. Performance Analysis of the Improved Algorithm

**Figure 8:**
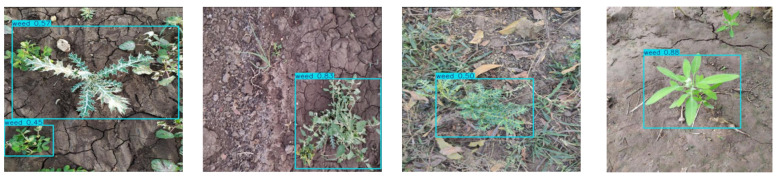
Detection results of YOLOv12n algorithm on partial test set.

**Figure 9:**
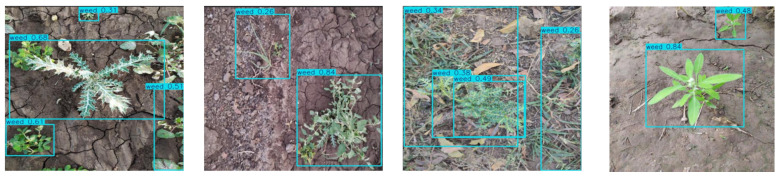
Detection results of improved YOLOv12n algorithm on partial test set.

We conducted experiments on the original and improved algorithms under identical conditions. Figure 8 and Figure 9 compare weed detection performance between the baseline and improved methods in actual sesame field scenarios. Figure 8 shows the detection results of the original YOLOv12n, while Figure 9 presents results from the improved YOLOv12-BDA. The comparison reveals that the improved algorithm more accurately identifies small weed targets, effectively reduces missed detections, and significantly improves bounding box positioning accuracy.

**Figure 10:**
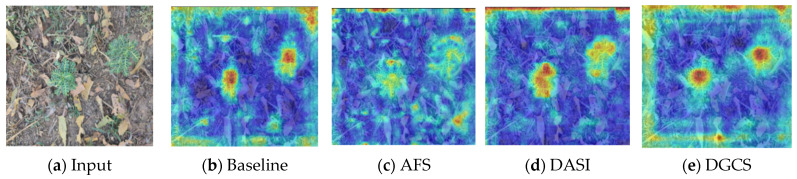
Input images and corresponding feature activation heatmaps of different improvement strategies.

To demonstrate the advantages of our approach, we analyzed the original images and their corresponding feature activation visualizations. Figure 10 shows the visualization results in the following order: (a) input images, (b) feature activation maps from the baseline YOLOv12, and (c–e) activation maps from the three improvement strategies (AFS, DASI, and DGCS).

Figure 10 reveals several key findings. The baseline YOLOv12 (b) identifies regions of interest but shows relatively sparse feature activations while producing background noise. The AFS module (c) significantly enhances activation intensity in target regions, with more concentrated activation patterns. The DASI module (d) improves spatial feature distribution, providing precise target localization while suppressing background noise. Finally, the DGCS module (e) maintains strong target activations while achieving clearer activation boundaries and more accurate alignment between target contours and activation regions.

The experiments in Section 3 demonstrate that YOLOv12-BDA consistently outperforms baseline models in detection accuracy and robustness. The improvements in mAP@50, mAP@50:95, and small target detection demonstrate the effectiveness of our proposed modules. However, beyond numerical results, it is important to examine the factors contributing to these gains, identify current limitations, and discuss implications for agricultural applications. These aspects are elaborated in Section 4.

## 4. Discussion

### 4.1. Performance Gain Analysis

Among the three proposed modules evaluated independently in ablation studies, the DASI (Dynamic Adaptive Scale-aware Interactive) module demonstrates the most substantial individual performance improvement. As shown in Table 5 (Group 4), DASI achieves a 10.00% improvement in mAP@50:95 (from 51.37% to 61.37%) and a 2.69% improvement in mAP@50 over the baseline YOLOv12n. This performance gain substantially exceeds those observed from individual implementation of AFS (0.32% improvement in mAP@50:95, Group 2) and DGCS (which exhibits performance degradation when implemented alone, Group 3). The superior performance of DASI can be attributed to its dynamic scale-adaptive weighting mechanism, which directly addresses the challenge of multi-scale feature fusion in agricultural scenarios where target scale distribution varies dramatically within individual images. The synergistic combination of all three modules (Group 8) ultimately achieves a cumulative 16.09% improvement in mAP@50:95, indicating complementary rather than redundant functionality across the proposed architectural innovations.

Each proposed module contributes uniquely to the overall performance improvement. Ablation studies (Table 5) reveal both the individual effects and synergistic interactions of the three key innovations. The DLU (Dynamic Learning Unit) module in the AFS dual-backbone architecture improved mAP@50 by 3.06% over the baseline YOLOv12n. This gain is primarily attributed to its ability to address information inconsistency between the two backbone branches. As shown in Figure 9, the DLU module enhances feature activation in the core regions of weed targets while maintaining computational efficiency through shared stem processing. The DLU module implements a dynamic weight generation strategy to adaptively fuse features from dual-backbones. While this approach shares conceptual similarities with attention-based adaptive weighting mechanisms originally developed for sequence modeling tasks [28,40], our implementation is specifically tailored for cross-branch feature alignment in computer vision applications. This design demonstrates adaptability in handling feature variability, which proves particularly beneficial in complex agricultural environments where lighting conditions and plant morphologies vary significantly.

The DGCS (Dynamic Grouped Convolution and Channel Mixing Transformer) module produced limited individual improvements (Group 3 in Table 5) but yielded substantial synergistic gains when combined with other modules. The 1:3 channel splitting ratio with grouped convolution design helps reduce computational load without compromising representational capability [41], which is particularly relevant for detecting small weeds in densely planted sesame fields. The DASI (Dynamic Adaptive Scale-aware Interactive) module achieved the largest single-module improvement among the three proposed components, increasing mAP@50 by 2.69% and mAP@50:95 by 10.00% (from 51.37% to 61.37%, Group 4 in Table 5). This 10.00% improvement in mAP@50:95 represents a substantially larger gain compared to the 0.32% improvement from AFS alone (Group 2) and the performance degradation observed with standalone DGCS implementation (Group 3), establishing DASI as the most impactful architectural modification for the agricultural weed detection task.

The superior performance of DASI stems from three key factors. First, by implementing dynamic weight adjustments based on input feature activation characteristics rather than predetermined fixed weights, DASI effectively alleviates the semantic gap between high-level and low-level feature maps, a persistent challenge in multi-scale object detection [26]. The adaptive weighting mechanism (α = sigmoid (m_i)) enables scale-dependent feature emphasis, where fusion weights are computed dynamically based on current-resolution activation patterns. Second, DASI’s design addresses a critical characteristic of agricultural weed detection: extreme intra-image scale variation. Field images simultaneously contain small early-stage weed seedlings that benefit primarily from high-resolution spatial features and larger mature weeds that require semantic context from low-resolution features. Traditional FPN architectures employing static fusion weights cannot adapt to this heterogeneous scale distribution, whereas DASI’s dynamic mechanism automatically adjusts fusion emphasis based on target characteristics inferred from feature activations. Third, the computational efficiency achieved through four-partition channel division (O(1) complexity with respect to channel dimension) enables substantial performance gains without prohibitive computational overhead, maintaining practical viability for real-time agricultural applications. This dynamic weighting mechanism [42] significantly improves multi-scale detection accuracy, addressing the fundamental challenge of identifying targets with extreme scale variation characteristic of agricultural field imagery.

### 4.2. Architectural Advantages and Limitations in Agricultural Context

The proposed architectural modifications demonstrate specific advantages for sesame field weed detection applications while simultaneously exhibiting inherent limitations that warrant acknowledgment and consideration for future research directions. The dual-backbone Adaptive Feature Selection (AFS) architecture incorporating the Dynamic Learning Unit (DLU) effectively addresses the fundamental trade-off between spatial resolution preservation—essential for accurate localization of small-scale weed targets—and semantic feature abstraction—necessary for robust classification under varying environmental conditions and imaging scenarios. This parallel processing strategy proves particularly effective in agricultural detection scenarios where targets exhibit extreme scale variation within individual images, enabling the network to simultaneously process early-stage seedlings that require preserved fine-grained spatial features for precise boundary delineation and mature plants that benefit from high-level semantic context for robust species discrimination. However, this architectural design incurs substantial computational costs, as evidenced by the 76.2% parameter increase (2.56 M to 4.51 M) and 69.8% increase in GFLOPs (6.3 to 10.7) compared to baseline YOLOv12n. The inference time increases to 8.840 ms (24.8% overhead), yet maintains real-time capability at 113 FPS—substantially exceeding typical agricultural robotics requirements (≥30 FPS). This accuracy-computational cost trade-off reflects a design choice prioritizing detection quality: the 16.09% mAP@50:95 improvement demonstrates substantial enhancement in localization precision essential for targeted herbicide application. While increased requirements may limit deployment on severely constrained embedded platforms, the architecture remains suitable for mainstream agricultural UAVs and ground robots where enhanced detection accuracy justifies the computational investment.

The 1:3 channel splitting strategy combined with grouped convolution addresses a fundamental challenge in agricultural weed detection: extracting discriminative features from visually similar crop-weed pairs that exhibit substantial morphological overlap during early growth stages. Standard convolutional operations apply uniform receptive fields across all input channels, processing spatial features identically regardless of the semantic content encoded within different channel subsets. This uniform processing paradigm potentially overlooks fine-grained inter-channel dependencies that prove essential for texture-based discrimination in scenarios where visual differences manifest primarily through subtle variations in surface characteristics rather than gross morphological distinctions. By channeling 25% of the feature map through intensive grouped convolution processing—where the number of convolution groups equals the channel count, creating an operation equivalent to depthwise separable convolution—while maintaining direct propagation pathways for the remaining 75% of channels, the DGCS module achieves dual complementary objectives. First, the subset subjected to grouped convolution undergoes detailed spatial feature extraction that captures local texture patterns including characteristics such as leaf venation density variations, edge sharpness distinctions, and surface smoothness differences that differentiate crop from weed foliage at fine spatial scales. Second, computational efficiency is maintained through selective processing that applies parameter-intensive operations to only a fraction of the channel dimension, enabling real-time inference capabilities suitable for deployment on resource-constrained agricultural platforms including unmanned aerial vehicles and ground-based autonomous robots [43].

The channel reshuffling operation implemented following the grouped convolution stage addresses a fundamental architectural limitation inherent in group-wise feature processing: the restriction of information flow within individual channel groups that prevents interaction between complementary visual cues encoded across different groups. Standard grouped convolution divides the input channel dimension into G distinct groups and applies separate convolution kernels to each group independently, thereby preventing cross-group feature interaction within individual convolutional layers. This architectural constraint means that features learned within one group—for example, color-related features capturing green intensity variations that correlate with chlorophyll content differences between species—cannot directly interact during convolution with features learned in separate groups, such as texture-related features encoding surface smoothness characteristics or shape-related features representing leaf margin regularity patterns [43]. By implementing a deterministic channel permutation pattern through tensor reshape and transpose operations, the reshuffling mechanism systematically redistributes channels across groups according to a predefined permutation function, enabling subsequent processing layers to access mixed feature representations that incorporate information from all original channel groups. This cross-group information exchange mechanism proves particularly relevant for agricultural weed detection applications, where discriminative features distinguishing morphologically similar species typically emerge from specific combinations of complementary visual cues distributed across different feature channels: color features reflecting species-specific pigmentation patterns in one subset of channels, texture features encoding surface morphology characteristics in another subset, and shape features capturing leaf margin properties in a third subset. By facilitating systematic interaction between these complementary feature types while maintaining the computational efficiency advantages of the asymmetric 1:3 channel processing strategy, the DGCS module achieves effective discrimination between visually similar crop and weed species without imposing prohibitive computational requirements that would preclude real-time deployment in practical agricultural operations.

The Dynamic Grouped Convolution and Channel Mixing Transformer (DGCS) module offers computational efficiency advantages through its asymmetric 1:3 channel splitting strategy, processing only 25% of channels through parameter-intensive grouped convolution operations while maintaining direct propagation pathways for the remaining 75%, thereby achieving substantial parameter reduction compared to conventional full-channel processing approaches. This selective processing design proves suitable for edge deployment scenarios where computational resources are constrained, such as battery-powered autonomous agricultural robots or unmanned aerial vehicles with limited onboard processing capabilities. However, this asymmetric channel allocation represents a fundamental design compromise: the aggressive reduction in the proportion of channels subjected to intensive processing may potentially discard discriminative features in detection scenarios characterized by extremely subtle inter-class variations. For example, distinguishing between sesame seedlings and Amaranthus species during early growth stages—when both species exhibit minimal morphological differentiation and share highly similar green spectral signatures, comparable leaf shapes, and equivalent spatial footprints—may require more comprehensive feature processing than the 25% allocation provides. The channel reshuffling mechanism partially addresses this limitation by enabling cross-group information exchange that allows features from different channel subsets to interact in subsequent processing layers. However, the effectiveness of this compensation strategy depends fundamentally on the assumption that systematic channel permutation patterns can adequately redistribute complementary discriminative features across groups, an assumption that may not hold uniformly across all agricultural imaging conditions and species combinations.

The Dynamic Adaptive Scale-aware Interactive (DASI) module achieves the most substantial individual performance improvement among the three proposed architectural components, demonstrating a 10.00% enhancement in mAP@50:95 (from 51.37% to 61.37%) when evaluated independently in ablation studies (Group 4, Table 5). This performance gain stems from the module’s dynamic weighting mechanism that computes fusion weights adaptively based on current-resolution feature activation patterns through the formulation α = sigmoid (m_i), enabling scale-dependent emphasis where different spatial locations within individual images receive optimized feature combinations tailored to local target characteristics. This adaptive fusion strategy effectively addresses the heterogeneous scale distribution characteristic of agricultural field imagery, where small early-stage weeds requiring high-resolution spatial features coexist spatially with large mature plants requiring semantic context. Nevertheless, the DASI module’s performance fundamentally depends on the assumption that current-resolution feature activation patterns provide reliable predictive signals for determining optimal fusion weights—an assumption that may be violated under extreme operational conditions poorly represented in training data distributions. Specifically, severe motion blur resulting from platform vibration during rapid unmanned aerial vehicle flight operations, dramatic illumination transitions during sunrise or sunset imaging periods, or dense vegetation occlusion creating complex shadow patterns may produce current-resolution activations that fail to accurately reflect the actual scale and semantic requirements of obscured or poorly imaged targets. Under such conditions, the dynamic weight generation mechanism may compute suboptimal fusion weights, potentially degrading detection performance below levels achievable with more robust static fusion strategies. Future research directions could investigate enhanced adaptive mechanisms that incorporate multi-modal sensor information—including time-of-day metadata, platform motion sensor data, or ambient illumination measurements—to improve the robustness of dynamic weight generation across the full spectrum of operational agricultural imaging conditions.

### 4.3. Comparison with Previous Studies

To contextualize our contributions within the broader landscape of agricultural weed detection research, we provide systematic comparison with recent YOLO-based detection systems across different crop types. Table 8 presents a comprehensive comparison of representative methods.

Zhou et al. [20] achieved the highest mAP@50 (95.3%) on cotton using context augmentation and selective kernel attention, but with higher computational cost (18.2 GFLOPs). Liu et al. [21] integrated MobileViTv3 with BiFPN for wheat detection (89.7% mAP@50), requiring 6.8 M parameters. Wang et al. [23] demonstrated parameter efficiency with transformer-based TIA-YOLOv5 (3.6 M parameters, 90.0% mAP@50).

The most relevant comparison is with Chen et al. [8], who also addressed sesame weed detection. Our method achieves 4.07% higher mAP@50 (91.57% vs. 87.5%) with lower computational cost (10.7 vs. 12.3 GFLOPs), demonstrating the effectiveness of our dual-backbone architecture. While Zhou et al.’s superior performance (95.3%) may partially reflect differences in dataset characteristics—CWD12 contains 5648 images across 12 weed classes, whereas our dataset focuses on binary crop-weed classification with 1300 images—the consistent improvements across methods validate the value of specialized architectures for agricultural applications.

Our DASI module differs from the ASFF used in prior work [8,23] through scale-adaptive dynamic weighting (α = sigmoid (m_i)) rather than static weight computation. This enables scale-dependent feature emphasis that adapts to local content, providing improved robustness to extreme scale variations where small weed seedlings (<32 × 32 pixels) and large mature plants coexist. Combined with our dual-backbone design that explicitly preserves spatial and semantic features through parallel pathways, YOLOv12-BDA achieves competitive balance between accuracy (91.57% mAP@50), efficiency (4.51 M parameters), and computational requirements (10.7 GFLOPs) for precision agriculture applications.

### 4.4. Limitations and Challenges

Several important limitations must be acknowledged regarding the scope and interpretation of our experimental validation. First, concerning the cross-domain transfer evaluation on the WeedCrop dataset (Section 3.5), it is essential to distinguish between cross-domain transfer and within-domain generalization. As detailed in Section 2.3.2, the substantial differences between the sesame and WeedCrop datasets—encompassing completely different crop and weed species, multiple imaging systems, and mixed environmental conditions—constitute a significant domain gap. The substantial performance degradation observed on WeedCrop (mAP@50:95 from 67.46% to 45.95%, a 21.51 percentage point reduction) quantitatively confirms this domain gap, indicating that this evaluation represents cross-task transfer rather than true cross-scene generalization. While the maintained relative performance advantage over baseline models demonstrates architectural robustness, this should not be interpreted as evidence that YOLOv12-BDA can generalize broadly across diverse agricultural scenarios without adaptation. True within-domain generalization would require evaluation on the same crop-weed species pairs across different geographical regions, seasons, and imaging conditions—systematically isolating environmental and operational factors while maintaining consistent biological targets. Comprehensive assessment of generalization capability would necessitate evaluation across multiple crop types (e.g., wheat, cotton, maize) with their respective weed species, diverse geographical regions representing different soil types and climate zones, and various growth stages throughout the cultivation cycle. The current two-dataset evaluation, while demonstrating architectural transferability, provides insights into design soundness rather than operational generalizability across the full spectrum of precision agriculture applications. Extension to multi-species weed identification tasks would provide additional evidence of scalability, as the current validation encompasses only binary crop-weed classification [44,45]. The training dataset sizes (1300 images for sesame, 2822 images for WeedCrop), while sufficient for demonstrating architectural improvements, represent a modest scale compared to large-scale agricultural datasets used in some recent studies.

Second, the experimental comparison encompasses YOLO-series architectures representing key evolutionary transitions (YOLOv5n through YOLOv12n) and RT-DETR as a transformer-based alternative, but does not extend to all recent lightweight detectors published in 2024–2025. This focused comparison strategy reflects methodological considerations for agricultural deployment contexts. The YOLO framework has demonstrated predominant adoption in precision agriculture, with documented implementations across diverse crop systems [8,20,21,23]. The selected baseline progression systematically captures fundamental architectural paradigm shifts: anchor-free detection (YOLOv8n), task-decoupled prediction heads (YOLOv10n), enhanced multi-scale feature extraction (YOLOv11n), and attention-centric design (YOLOv12n). RT-DETR provides comparison with transformer-based detection employing fundamentally different object query mechanisms. Furthermore, backbone comparison experiments (Table 3) establish architectural diversity through evaluation against efficiency-optimized designs (FasterNet, StarNet), neural architecture search strategies (EfficientNet-B0), and hybrid CNN-Transformer architectures (EfficientViT). Nevertheless, the absence of emerging detection architectures employing alternative design principles—such as efficient instance matching (DEIM) or dynamic feature integration networks (DFINE)—represents a limitation. Future work will incorporate comprehensive evaluation against emerging lightweight detectors to establish generalizability beyond YOLO-based architectures.

Third, computational requirements remain a consideration for deployment on resource-limited agricultural devices [46,47]. Inference efficiency analysis shows that YOLOv12-BDA achieves 8.840 ms inference time, representing a 24.8% increase compared to YOLOv12n’s 7.085 ms baseline performance. This inference overhead results from the dual-backbone architecture and dynamic modules (DLU, DGCS, DASI). While this latency remains suitable for typical UAV-based agricultural monitoring at 2–5 Hz capture rates, achieving efficient real-time performance on embedded systems (e.g., ARM-based processors) commonly used in agricultural machinery requires further optimization through techniques such as model pruning, quantization, or knowledge distillation to reduce deployment footprint while maintaining detection accuracy.

Fourth, robustness under diverse operational conditions represents an important consideration for practical deployment. While our experimental evaluation demonstrates performance improvements on standard test conditions, several challenging scenarios require acknowledgment. Performance under extreme conditions—such as severe occlusion (>60% boundary obscurement), extremely dense weed coverage, or adverse weather—has not been systematically quantified. Agricultural field environments exhibit substantial variability including illumination variations due to solar angle changes (early morning/late afternoon with long shadows), motion blur from platform movement or wind-induced vegetation motion, and varying crop maturity stages creating diverse target size distributions. Qualitative analysis suggests that the dual-backbone AFS architecture and dynamic DASI fusion provide robustness advantages under moderate challenge conditions through parallel feature processing and adaptive weighting. However, controlled dataset settings may not fully reflect operational variability in real farming environments, where combinations of multiple challenge factors (e.g., severe occlusion under low-light conditions) may compound detection difficulty. Systematic field trials with explicit documentation of environmental conditions and controlled manipulation of individual challenge factors would be necessary to establish reliable performance boundaries and operational guidelines. Additionally, current work focuses on binary crop-weed classification without targeting individual weed species. Species-level identification would be essential for precision herbicide application [48,49] and could further increase the system’s practical value. Future work will expand dataset diversity to include multiple crops and environmental conditions, optimize the model for embedded and low-power hardware through model compression techniques, evaluate robustness under extreme operational scenarios with systematic challenge factor manipulation, and explore domain adaptation methods to enhance cross-crop generalization.

### 4.5. Implications for Smart Agriculture

The improved YOLOv12-BDA algorithm shows strong potential for integration into advanced smart farming systems. Its 91.57% mAP@50 performance, combined with real-time processing capability, makes it suitable for deployment on autonomous agricultural platforms such as unmanned aerial vehicles (UAVs) and ground-based robots for in-field weed monitoring and precision management [50]. The successful deployment of YOLO-based detection systems on UAV platforms has been demonstrated in recent agricultural applications, including automated rice seedling counting and localization from aerial imagery [51], confirming the technical feasibility and operational effectiveness of integrating deep learning models with aerial monitoring systems. Similarly, hybrid deep learning architectures combining segmentation and classification networks have achieved robust performance in agricultural product quality assessment tasks [52], demonstrating the versatility of deep learning frameworks across diverse precision agriculture applications. The modular architecture also facilitates integration into agricultural IoT frameworks, enabling combination with other sensor systems (e.g., soil moisture, nutrient level, microclimate monitoring). This could form the basis for comprehensive decision-support systems that combine weed detection with crop health assessment, delivering targeted management recommendations.

In practical implementation, coupling our detection results with variable-rate herbicide application systems [49,53] can enable precise, localized treatments, substantially reducing chemical usage while ensuring weed control efficiency. This aligns with economic and environmental sustainability goals in modern agriculture, where reducing pesticide inputs while maintaining crop productivity represents a critical challenge. The system’s real-time processing capability also supports the development of autonomous weeding robots that can operate continuously during optimal treatment windows.

Finally, the adaptability of our architecture suggests strong potential for transfer learning applications [54], allowing rapid customization for new crop-weed detection tasks with minimal retraining data. Such scalability could accelerate the adoption of AI-enabled weed detection across diverse agricultural settings worldwide, contributing to the broader digitalization of agricultural practices and supporting food security objectives in the face of growing global demand.

## 5. Conclusions

This study addresses the challenge of weed detection in sesame fields by developing YOLOv12-BDA, a dynamic multi-scale architecture that enhances detection accuracy while maintaining real-time processing capabilities. The proposed method introduces three key innovations that work synergistically to improve upon limitations in existing approaches.

The Adaptive Feature Selection (AFS) dual-backbone architecture incorporating the Dynamic Learning Unit (DLU) module effectively enhances cross-branch feature extraction capabilities while reducing computational redundancy. The replacement of the C3K2 component with the novel Dynamic Grouped Convolution and Channel Mixing Transformer (DGCS) module substantially improves real-time detection performance for small weed targets in complex agricultural backgrounds. The integration of the Dynamic Adaptive Scale-aware Interactive (DASI) module into the neck network successfully addresses semantic information propagation limitations in traditional feature pyramid networks, enabling superior multi-scale detection accuracy.

Experimental validation on the sesame weed detection dataset demonstrates performance improvements: YOLOv12-BDA achieves mAP@50 and mAP@50:95 of 91.57% and 67.46%, respectively, representing improvements of 4.67% and 16.09% over the baseline YOLOv12n model. Comparative analysis shows improved performance over YOLOv5n, YOLOv8n, YOLOv10n, and YOLOv11n, with mAP@50 improvements of 6.43%, 11.72%, 7.15%, and 5.33%, respectively. Ablation studies indicate that each proposed module contributes to overall performance, with the DASI module providing the most substantial individual improvement (10% increase in mAP@50:95).

The improved algorithm shows effectiveness in detecting small weed targets while reducing false negative rates on the evaluated datasets. Feature activation visualizations indicate enhanced target localization and reduced background interference compared to baseline methods.

This research contributes to the advancement of intelligent weed management systems. The demonstrated improvements in detection accuracy—particularly the 16.09% enhancement in mAP@50:95—indicate potential for deployment on autonomous agricultural platforms where detection quality is prioritized. While the architecture incurs increased computational requirements (76.2% parameter increase), it maintains real-time capability (113 FPS), supporting precision herbicide application systems where enhanced detection translates to reduced chemical usage. However, practical implementation would require further validation across diverse agricultural environments and adaptation to specific crop-weed systems to ensure operational robustness.

Cross-domain transfer evaluation on the WeedCrop Image Dataset provides evidence of architectural robustness under substantial domain shift. Despite a 21.51 percentage point reduction in mAP@50:95 due to fundamental differences in species composition, imaging systems, and environmental conditions, YOLOv12-BDA maintained relative improvements of 6.44% and 9.11% in mAP@50 and mAP@50:95 over baseline YOLOv12n. This preservation of relative advantage suggests that the proposed architectural modifications provide fundamental feature extraction capabilities that partially transfer across different agricultural detection tasks. However, the substantial absolute performance reduction indicates that practical deployment to new crop systems would require domain-specific adaptation and validation. Comprehensive evaluation across diverse crop types, geographical regions, and environmental conditions would be necessary to establish broader generalization capabilities for precision agriculture applications.

Future work will focus on extending validation to additional crop varieties and geographical regions, optimizing the architecture for embedded hardware deployment through model compression techniques, investigating the model’s performance on multi-species weed identification tasks, and developing domain adaptation strategies to facilitate transfer to new crop-weed systems. These efforts will strengthen evidence for broader agricultural applicability while addressing current limitations in dataset diversity and computational efficiency.

## Figures and Tables

**Table 1:** Data Augmentation Configuration.

Category	Parameter	Value	Purpose
Color Space	hsv_h	0.015	Hue variation (±1.5%)
	hsv_s	0.700	Saturation variation (±70%)
	hsv_v	0.400	Brightness variation (±40%)
Geometric	translate	0.1	Spatial translation (±10%)
	scale	0.5	Scale range (0.5×–1.5×)
	fliplr	0.5	Horizontal flip probability
	mosaic	1.0	Four-image composition
	close_mosaic	10	Mosaic disable (final epochs)
Occlusion	auto_augment	randaugment	Automated augmentation
	erasing	0.4	Random erasing probability

Note: Parameters follow YOLOv12 framework conventions. Disabled augmentations (rotation = 0.0, shear = 0.0, perspective = 0.0, flipud = 0.0, bgr = 0.0, mixup = 0.0) are omitted from the table.

**Table 2:** Performance Comparison of Various YOLO Models.

Models	Precision (%)	Recall (%)	mAP@50 (%)	mAP@50:95 (%)	Inference Time (ms)	Parameters (M)	GFLOPS
RT-DETR	84.23	81.15	83.35	45.96	12.027	19.87	56.9
YOLO v5n	80.82	85.35	85.14	51.96	4.771	2.50	7.1
YOLO v8n	81.26	69.62	79.85	40.52	4.892	3.01	8.1
YOLO v10n	86.15	81.01	84.42	51.82	5.260	2.70	8.2
YOLO v11n	83.85	85.41	86.24	51.51	5.939	2.58	6.3
YOLOv12n	86.95	84.31	86.90	51.37	7.085	2.56	6.3
YOLOv12-BDA	85.36	87.60	91.57	67.46	8.840	4.51	10.7

**Table 3:** Comparison of Different Trunks.

Models	Precision (%)	Recall (%)	mAP@50 (%)	mAP@50:95 (%)	Inference Time (ms)	Parameters (M)	GFLOPS
YOLO12-AFS	84.23	87.91	89.96	51.69	7.046	4.55	9.4
YOLO12-timm	84.18	83.54	85.47	49.87	8.038	12.44	33.0
YOLO12-fasternet	85.55	86.08	86.55	51.18	7.561	3.41	8.6
YOLO12-starnet	74.81	86.61	86.33	50.00	5.534	1.54	4.5
YOLO12-efficientViT	82.65	83.54	87.12	50.28	11.372	3.31	7.4

**Table 4:** Performance Comparison of Different Upsampling Modules in Neck Network. All models are based on YOLOv12n architecture with only the upsampling module being varied.

Models	Precision (%)	Recall (%)	mAP@50 (%)	mAP@50:95 (%)	Inference Time (ms)	Parameters (M)	GFLOPS
YOLO12-DySample	88.62	84.81	87.75	50.73	8.63	2.55	6.3
YOLO12-CARAFE	83.91	85.83	85.55	50.85	12.57	2.68	6.6
YOLO12-EUCB	85.27	87.94	88.37	52.42	8.79	2.62	6.7
YOLO12-DASI	89.40	88.74	89.59	61.37	9.42	2.62	7.4

**Table 5:** Ablation study of the improved modules. The “√” sign indicates that the module was added to the YOLOv12n network.

Group	AFS	DGCS	DASI	Precision (%)	Recall (%)	mAP@50(%)	mAP@50:95(%)	Inference Time (ms)
1				86.95	84.31	86.9	51.37	7.085
2	√			84.23	87.91	89.96	51.69	7.046
3		√		51.88	67.11	66.07	32.23	6.853
4			√	89.4	88.74	89.59	61.37	9.422
5	√	√		89.81	86.08	90.52	51.24	7.194
6	√		√	90.24	82.89	90.41	59.02	10.156
7		√	√	80.19	77.63	85.45	50.54	5.493
8	√	√	√	85.36	87.60	91.57	67.46	8.840

**Table 6:** Learning Rate Sensitivity Analysis for YOLOv12-BDA.

Learning Rate	Precision (%)	Recall (%)	mAP@50 (%)	mAP@50:95 (%)
0.001	79.46	82.55	85.67	52.97
0.010	85.36	87.60	91.57	67.46
0.015	79.91	84.01	85.74	56.22
0.020	88.29	77.28	86.54	55.91
0.100	85.31	81.59	88.52	56.90

**Table 7:** Cross-Domain Transfer Performance Comparison on WeedCrop Dataset.

Models	Precision (%)	Recall (%)	mAP@50 (%)	mAP@50:95 (%)	Inference Time (ms)	Parameters (M)	GFLOPS
YOLO v5n	61.15	63.05	61.08	29.62	5.393	2.18	5.8
YOLO v8n	66.28	70.16	66.51	31.27	4.348	2.68	6.8
YOLO v10n	67.28	64.20	68.52	37.85	6.019	2.27	6.5
YOLO v11n	69.76	72.01	72.59	36.82	7.338	2.58	6.3
YOLOv12n	71.54	73.41	73.21	36.84	8.095	2.51	5.8
YOLOv12-BDA	72.87	77.85	79.65	45.95	10.532	4.51	10.7

**Table 8:** Comparison with Recent YOLO-based Agricultural Weed Detection Methods.

Study	Method	Crop Type	mAP@50 (%)	Parameters (M)	GFLOPS	Key Innovation
Zhou et al. [20]	YOLO-ACE	Cotton	95.3	5.2	18.2	Context augmentation + attention
Liu et al. [21]	YOLOv8-MobileViT	Wheat	89.7	6.8	15.6	MobileViTv3 + BiFPN + MPDIoU
Wang et al. [23]	TIA-YOLOv5	Multi-crop	89.2	3.6	7.4	Transformer + CFFI + ASFF
Chen et al. [8]	YOLO-sesame	Sesame	87.5 *	4.1	12.3	Attention (LIP + SE) + ASFF
Ours	YOLOv12-BDA	Sesame	91.57	4.51	10.7	Dual-backbone + dynamic fusion

Note: * Chen et al. reported F1 scores (F1-sesame: 0.91, F1-weed: 0.92) and overall mAP of 96.16%. The mAP@50 value of 87.5% is estimated by converting their F1 scores using the relationship between precision, recall, and AP for binary classification tasks.

## Data Availability

The original contributions presented in the study are included in the article. Further inquiries can be directed to the corresponding author.
